# Supplementary material for: Reversal of DDK-Mediated MCM Phosphorylation by Rif1-PP1 Regulates Replication Initiation and Replisome Stability Independently of ATR/Chk1
Source: Cell Rep. 2017 Mar 7;18(10):2508–20. doi: 10.1016/j.celrep.2017.02.042 (PMC5357733; doi:10.1016/j.celrep.2017.02.042)
Supplement: Document S1. Figures S1–S6 [file mmc1.pdf]

Cell Reports, Volume *18*

Supplemental Information

Reversal of DDK-Mediated MCM Phosphorylation by Rif1-PP1 Regulates Replication Initiation and Replisome Stability Independently of ATR/Chk1

Robert C. Alver, Gaganmeet Singh Chadha, Peter J. Gillespie, and J. Julian Blow

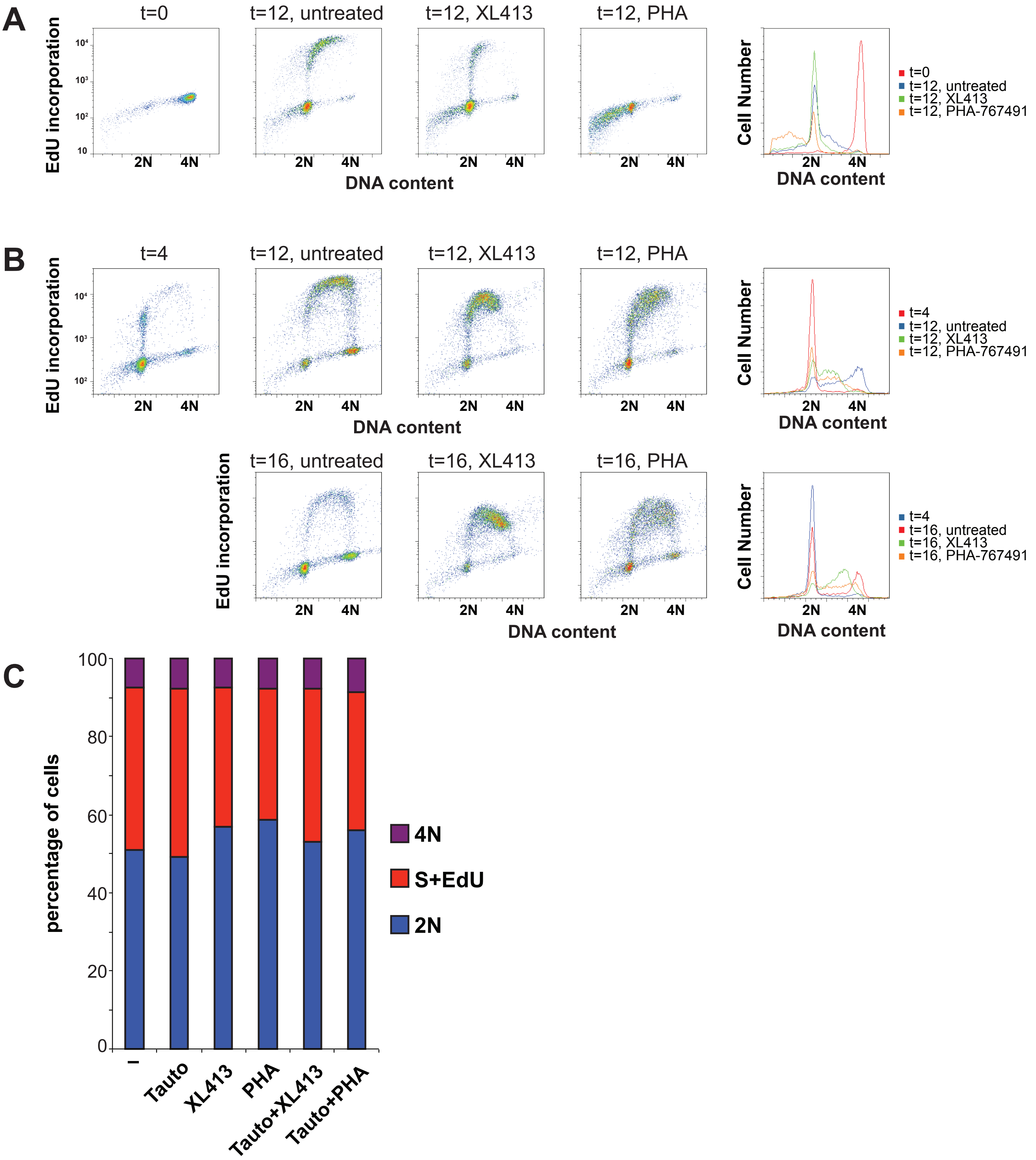

Figure S1. Effect of PP1 and Cdc7 inhibitors on the cell cycle. ***Related to Figure 2***

A-B, HeLa cells were synchronized by mitotic shakeoff and were (A) immediately treated for 12 hrs with 10 µM PHA-767491 or 10 µM XL413 or (B) treated with the same concentration of Cdc7 inhibitor for 8 or 12 hours after allowing cells to move into early S phase. Cells were pulsed with EdU for 30 min and analysed by flow cytometry. 2D EdU/DNA content plots are shown for each treatment. Frequency plots of DNA content are shown on the right. Note that the plots have not had the sub-2N population gated out.

C, HeLa cells were synchronized by mitotic shakeoff, allowed to move into early S phase and then subsequently treated for 2 hours with 225 nM tautomycetin, 10 µM XL413, or 10 µM PHA-767491 in the indicated combinations. The relative populations of cells in each cell cycle phase were quantified by 2D flow cytometry.

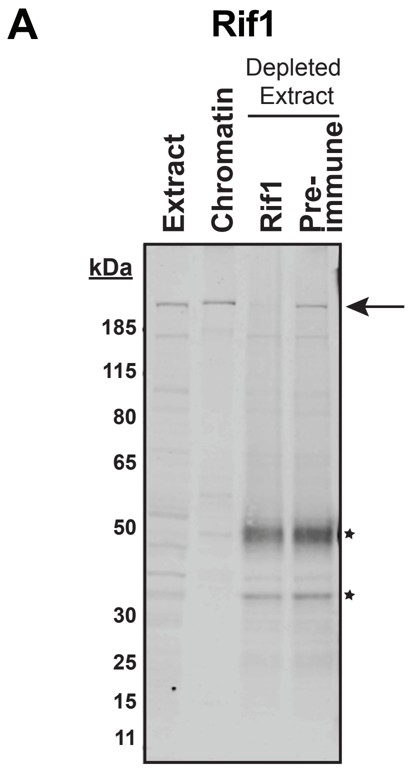

B

Xenopus

Protein Description	Peptide Count	
	Rif1	IgG
1. Rif1 protein	81	0
2. Pcm-1 protein; Pericentriolar material 1 protein	52	0
3. Nucleoprotein TPR (Fragment)	19	0
4. plectin-like isoform X3	6	0
5. Ataxia telangiectasia mutated (Fragment); ATM	3	0
6. CAP-GLY domain containing linker protein 1	3	0
7. Haspin	3	0

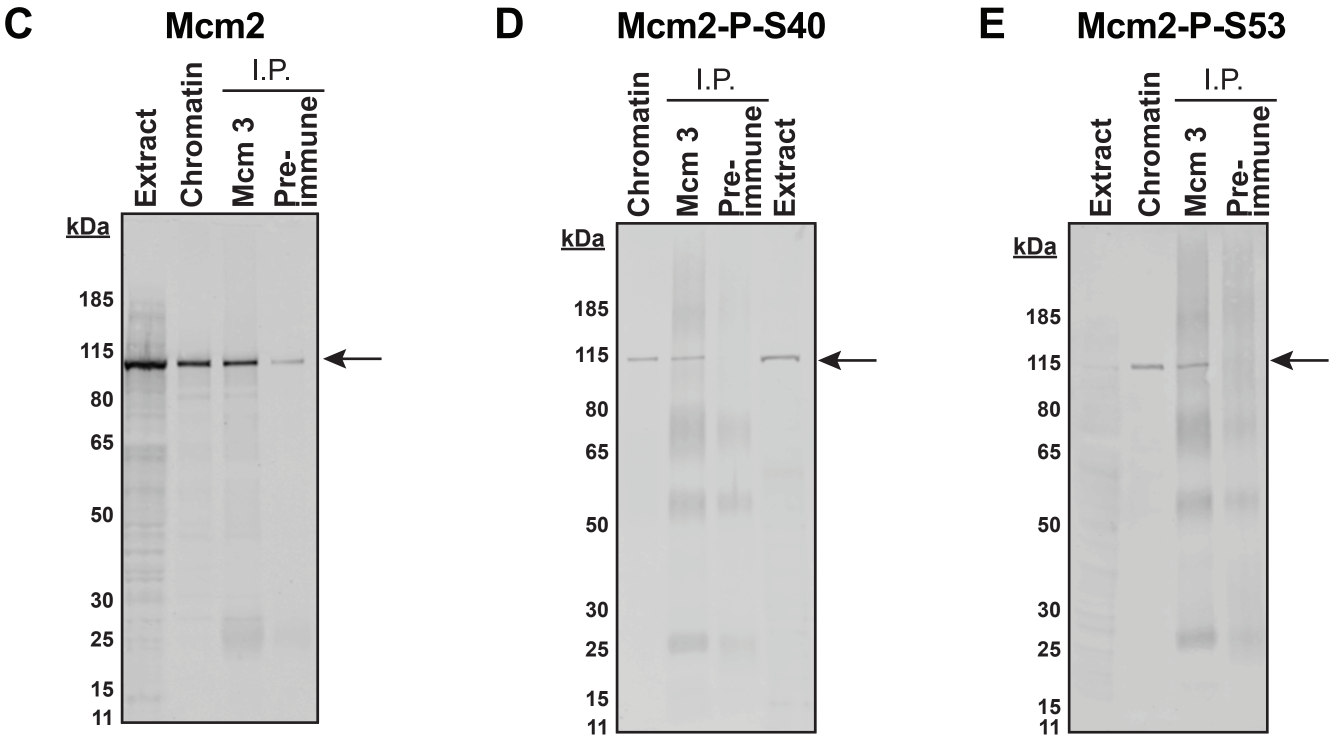

Figure S2. **Characterisation of antibodies in Xenopus.** ***Related to Figure 3***

A, 0.5 µl Xenopus egg extract, chromatin isolated from early S phase (40 minutes) and 0.5 µl extract depleted using antibodies against Rif1 or pre-immune IgGs were separated by SDS-PAGE and immunoblotted with the Xenopus Rif1 antibody. **B**, Immunoprecipitations (IP) from Xenopus egg extract using Rif1 antibody or control rabbit IgG (Sigma) covalently coupled to Protein-A Sepharose beads. IP samples were run on a 4–12% gradient NuPAGE gel (Invitrogen). The gel was stained with SimplyBlue SafeStain (Invitrogen). Bands at the size of Rif1 were cut from both Rif1 and IgG lanes, samples were reduced with dithiothreitol, alkylated with iodoacetamide and in-gel digested with trypsin. The extracted peptide solutions were analysed using nano LC-MS/MS on an LTQ Orbitrap Velos (ThermoFisher, San Jose, CA). The top five hits by peptide number for proteins identified only in Rif1 IP and not in control IgG IP are presented. **C-E**, 0.5 µl Xenopus egg extract, chromatin isolated from early S phase (40 minutes) and proteins immunoprecipitated using Xenopus Mcm3 or pre-immune serum were separated by SDSPAGE and immunoblotted with the Xenopus Mcm2 (C), Mcm2-P-S40 (D) and Mcm2-P-53 (E) antibody. The migration of molecular weight markers (sizes in kDa) is shown to the left.

Xenopus

A

Protein Description	Peptide Count	
	**Rif1**	**IgG**
Rif1 protein	48	0
Serine/threonine-protein phosphatase PP1-beta catalytic subunit;ppp1cb	3	0

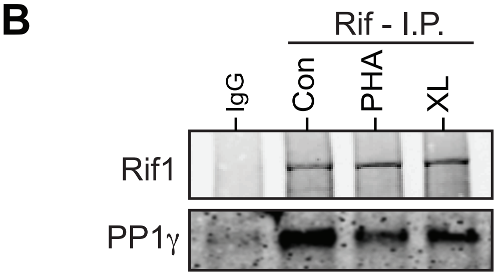

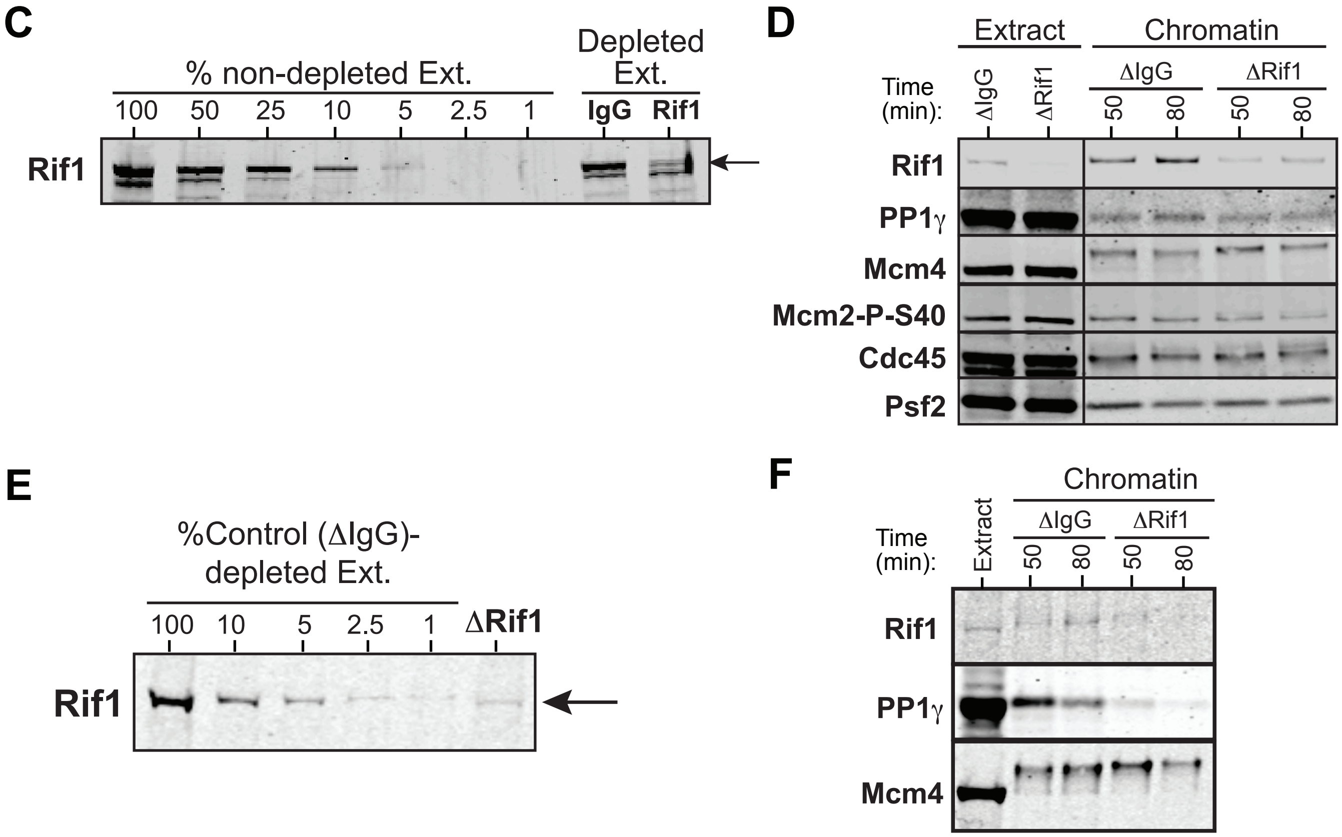

Figure S3. Characterisation of Rif1 depletion in Xenopus egg extract. ***Related to Figure 3***
A, Chromatin was isolated from Xenopus egg extract in mid S-phase. Immunoprecipitations (IP) using Xenopus Protein-A-purified Rif1 antibody or control non-immune IgG coupled to Protein-A Sepharose was carried out as described (Gillespie et al. 2012). The samples were reduced with dithiothreitol, alkylated with iodoacetamide and digested with trypsin. The extracted peptide solutions were analysed using nano LC-MS/MS on an LTQ Orbitrap Velos (ThermoFisher, San Jose, CA). The top hits by peptide number for proteins identified only in Rif1 IP and not in control IgG IP are presented. **B**, Xenopus egg extract were optionally supplemented and incubated with PHA-767491 or XL413 for 45 min before Rif1 was immunoprecipitated from extracts using antibodies against Rif1 or with pre-immune rabbit IgG. The samples were immunoblotted for Rif1 and PP1γ. **C-F**, Xenopus egg extract was immunodepleted with either nonimmune IgG or Rif1 antibodies. Two different extent of depletions are shown (C,D and E,F). So that the efficiency of depletion could be estimated, 0.5 μl of each of the depleted extracts and known amounts of control depleted extract was immunoblotted for Rif1. The efficiency of immunodepletion was >90% (C) and >95% (E). D, F, Control (ΔIgG) and Rif1 (ΔRif1) depleted Xenopus extracts from C and E were incubated with demembranated sperm nuclei. Chromatin was isolated at indicated times and samples were immunoblotted for Rif1, Mcm4, Mcm2-P-S40, Cdc45, Psf2, and PP1γ.

Xenopus

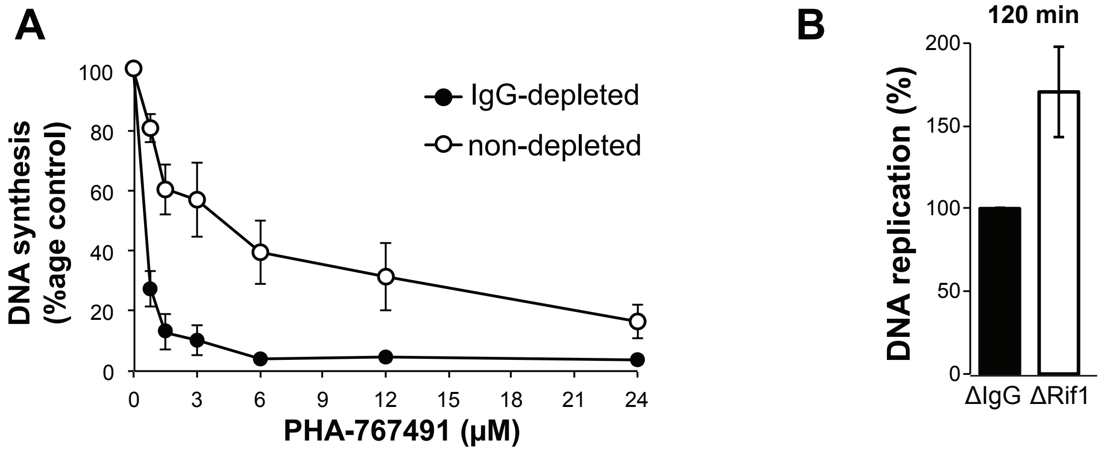

Figure S4. DNA replication upon Rif1 depletion in Xenopus egg extract. ***Related to Figure 3 & 4***
A, Control (non-depleted) or IgG-depleted Xenopus extracts were incubated with 3 ng/μl sperm DNA, [α-^{32}P]dATP and increasing concentration of PHA-767491. Total DNA synthesis at 240 min is expressed as a percentage of the synthesis in the absence of PHA-767491. Mean and S.E.M. of three independent experiments is shown. **B**, Control (Δ IgG) and Rif1 (ΔRif1) depleted Xenopus extracts were incubated with demembranated sperm nuclei and [α-^{32}P]dATP. Total DNA synthesis at 120 min is expressed as a percentage of the synthesis in control (ΔIgG-depleted) extract. Mean and S.E.M. of three independent experiments is shown.

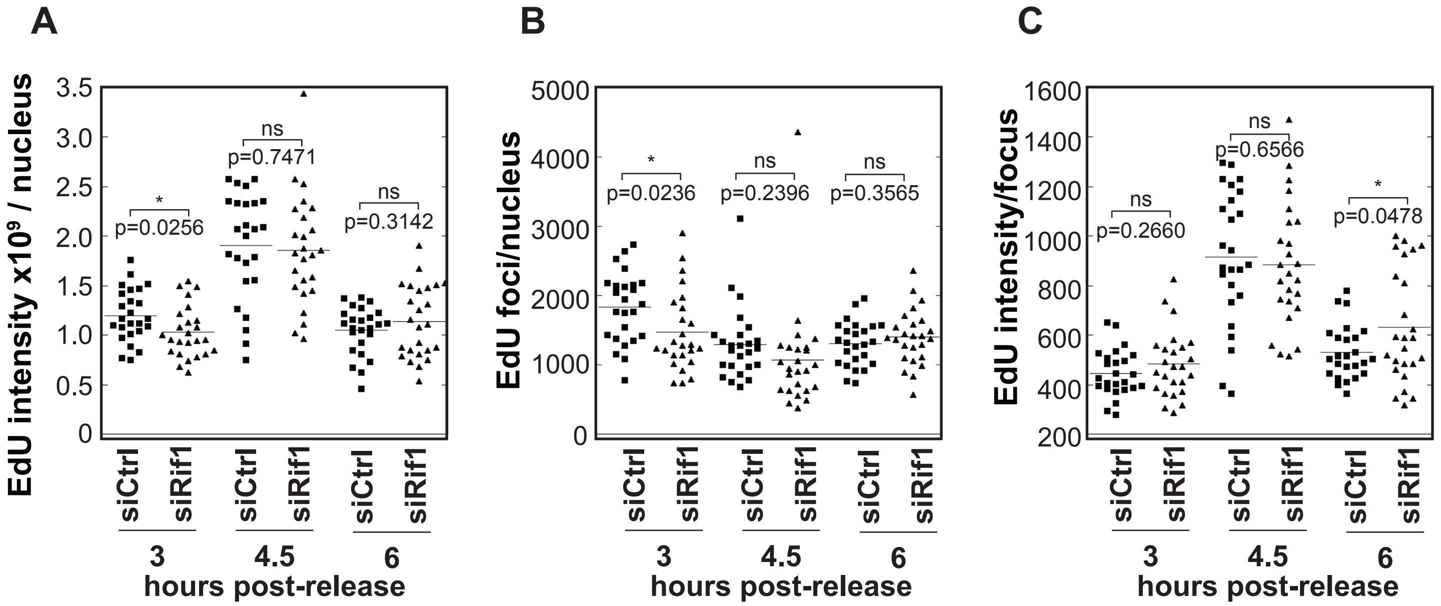

Figure S5. Quantification of the 3, 4.5, and 6 hr timepoints from Figure 5
HeLa cells were treated with control or Rif1 siRNA, synchronized by double thymidine block and then released. At the indicated periods after release cells were pulsed with EdU for 30 min. The spatial patterns of DNA synthesis were examined by fluorescence microscopy. At the indicated times after release the total EdU intensity of the nucleus (**A**), the number of replication foci per nucleus (**B**) and the intensity of the EdU foci (**C**) were quantified (25 nuclei/condition).

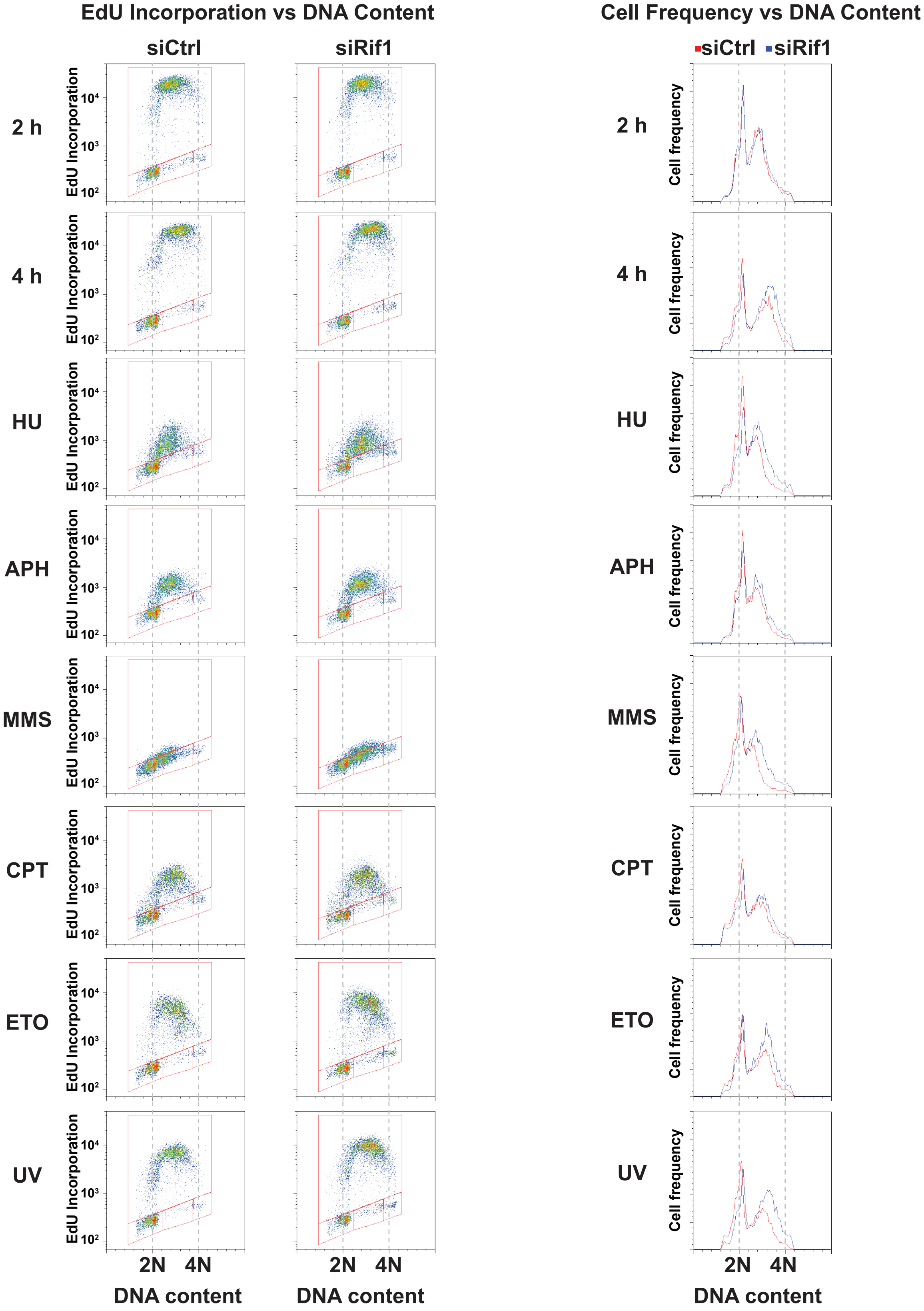

Figure S6. Rif1 regulates S phase progression and replisome stability. ***Related to Figure 7***
This is the same experiment as Fig. 7C. HeLa cells were treated control or Rif1 siRNA, synchronized by double thymidine block, and released 2 hrs before either no treatment or after the addition of hydroxyurea (5 mM), aphidicolin (1 mg/mL), methyl methane-sulfonate (0.02%), camptothecin (5 mM) etoposide (5 mM), or treating cells with UV irradiation (~45 J/m^2). Chemical treatments and recovery from irradiation lasted two hours before cells were harvested, fixed and subsequently analysed by flow cytometry. 2D EdU/DNA content plots are shown for each treatment. Frequency plots of DNA content are shown on the right.
