# Supplementary material for: Reversal of DDK-Mediated MCM Phosphorylation by Rif1-PP1 Regulates Replication Initiation and Replisome Stability Independently of ATR/Chk1
Source: Cell Rep. 2017 Mar 7;18(10):2508–20. doi: 10.1016/j.celrep.2017.02.042 (PMC5357733; doi:10.1016/j.celrep.2017.02.042)
Supplement: Document S2. Article plus Supplemental Information [file mmc2.pdf]

Cell Reports

Article

Reversal of DDK-Mediated MCM Phosphorylation by Rif1-PP1 Regulates Replication Initiation and Replisome Stability Independently of ATR/Chk1

Graphical Abstract

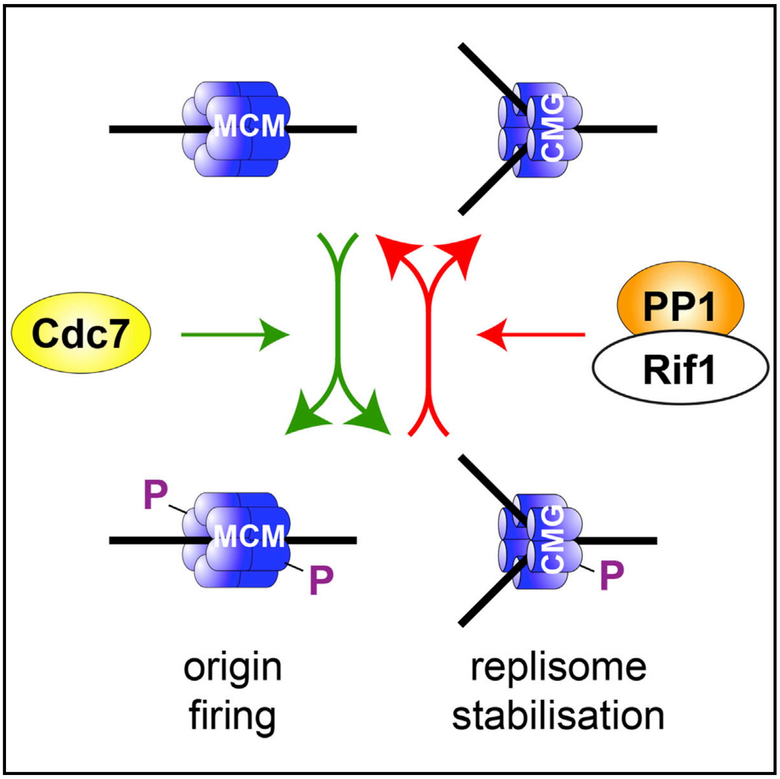

Highlights

- DDK-dependent phosphorylation of Mcm4, but not Mcm2, tracks with DNA replication
- DDK-inhibitors, PHA-767491 and XL-413, differentially affect MCM phosphorylations
- Vertebrate Rif1 targets PP1 to replication origins that reverse MCM phosphorylation
- Replisomes are destabilized by Rif1/PP1-mediated dephosphorylation

Authors

Robert C. Alver,
Gaganmeet Singh Chadha,
Peter J. Gillespie, J. Julian Blow

Correspondence

j.j.blow@dundee.ac.uk

In Brief

Alver et al. find that DDK-dependent MCM2-7 phosphorylation is opposed by Rif1-targeted PP1. Lack of Rif1 increases MCM phosphorylation and abolishes the ability to block origin firing, even when undergoing replication stress. In the absence of CHK1, the stability of stalled replisomes is severely compromised upon Rif-PP1-mediated dephosphorylation.

Alver et al., 2017, Cell Reports *18*, 2508–2520
March 7, 2017 © 2017 The Authors.
http://dx.doi.org/10.1016/j.celrep.2017.02.042

CrossMark

CellPress

OPEN ACCESS
CellPress

Cell Reports
Article

Reversal of DDK-Mediated MCM Phosphorylation by Rif1-PP1 Regulates Replication Initiation and Replisome Stability Independently of ATR/Chk1

Robert C. Alver,[1,2,3] Gaganmeet Singh Chadha,[1,2] Peter J. Gillespie,[1] and J. Julian Blow[1,4,*]
[1]Centre for Gene Regulation & Expression, School of Life Sciences, University of Dundee, Dundee DD1 5EH, UK
[2]Co-first author
[3]Present address: Division of Oncology Research, Mayo Clinic, Rochester, MN 55905-0002, USA
[4]Lead Contact
*Correspondence: j.j.blow@dundee.ac.uk
http://dx.doi.org/10.1016/j.celrep.2017.02.042

SUMMARY

Dbf4-dependent kinases (DDKs) are required for the initiation of DNA replication, their essential targets being the MCM2-7 proteins. We show that, in *Xenopus laevis* egg extracts and human cells, hyper-phosphorylation of DNA-bound Mcm4, but not phosphorylation of Mcm2, correlates with DNA replication. These phosphorylations are differentially affected by the DDK inhibitors PHA-767491 and XL413. We show that DDK-dependent MCM phosphorylation is reversed by protein phosphatase 1 (PP1) targeted to chromatin by Rif1. Loss of Rif1 increased MCM phosphorylation and the rate of replication initiation and also compromised the ability of cells to block initiation when challenged with replication inhibitors. We also provide evidence that Rif1 can mediate MCM dephosphorylation at replication forks and that the stability of dephosphorylated replisomes strongly depends on Chk1 activity. We propose that both replication initiation and replisome stability depend on MCM phosphorylation, which is maintained by a balance of DDK-dependent phosphorylation and Rif1-mediated dephosphorylation.

INTRODUCTION

The ordered assembly of replication proteins onto chromatin is highly regulated to ensure that DNA is precisely copied only once each cell cycle. Beginning in late M/early G1 phase, double hexamers of Mcm2–7 are loaded in excess onto replication origins. In S phase, Dbf4-dependent Cdc7-kinase (DDK) and cyclin-dependent kinase (CDK) promote the conversion of Mcm2-7 into active Cdc45-MCMs-GINS (CMG) replicative helicases.

DDK is an essential and conserved serine/threonine kinase that is formed of a Cdc7 catalytic subunit associated with its regulatory partner, Dbf4. In vertebrates, a second regulator of Cdc7, Drf1, has been identified (Montagnoli et al., 2002; Yanow et al., 2003; Takahashi and Walter, 2005; Yoshizawa-Sugata et al., 2005). The key target of DDK for the initiation of DNA replication is the Mcm2–7 complex. DDK phosphorylates the N terminus of several Mcm2–7 subunits (minichromosome maintenance proteins, or MCMs), specifically Mcm2, Mcm4, and Mcm6, (Lei et al., 1997; Sato et al., 1997; Jiang et al., 1999; Masai et al., 2000; Jares and Blow, 2000; Sheu and Stillman, 2010). Although the contribution of each of these phosphorylation events to initiation is not well understood, a key consequence of the hyper-phosphorylation of Mcm4 is the relief of an inhibitory signal that prevents replication (Sheu and Stillman, 2006, 2010). Significantly, cells lacking this Mcm4 N-terminal tail no longer require DDK activity to complete replication. There is evidence that Mcm2 phosphorylation by DDK is also required for DNA replication (Hardy et al., 1997; Bruck and Kaplan, 2015). In *Xenopus laevis* egg extracts, Mcm4 hyper-phosphorylation by DDK occurs primarily on chromatin (Poh et al., 2014), though experiments in mammalian cells have suggested that DDK phosphorylation of Mcm2 occurs primarily when it is not bound to DNA (Montagnoli et al., 2006).

Recent studies have revealed that protein phosphatase 1 (PP1) restrains DDK activity by reversing the Cdc7-dependent phosphorylation of Mcm4 (Poh et al., 2014; Hiraga et al., 2014; Davé et al., 2014; Mattarocci et al., 2014). In *Xenopus* egg extracts, DDK executes its essential function early in S phase by phosphorylating Mcm4 at all replication origins (Poh et al., 2014). However, PP1 continually removes these Mcm4 phosphorylations so that when DDK activity is inhibited in the middle of S phase, PP1 dephosphorylates MCMs at origins that have not yet fired, and replication fails to finish. In addition, induction of cell-cycle checkpoints resulted in increased PP1 recruitment and subsequent Mcm4 dephosphorylation without altering DDK activity (Poh et al., 2014).

In *Saccharomyces cerevisiae* and *Schizosaccharomyces pombe*, PP1 is targeted to MCMs by Rap1-interacting factor (Rif1) (Hiraga et al., 2014; Davé et al., 2014; Mattarocci et al., 2014). Loss of this interaction leads to premature Mcm4 phosphorylation and misregulation of origin firing and allows cells to compensate for reduced DDK activity. Loss of *S. pombe* or mammalian Rif1 deregulates the replication timing program (Cornacchia et al., 2012; Hayano et al., 2012; Yamazaki et al., 2012). These findings are consistent with the hypothesis that Rif1 negatively regulates DNA replication by recruiting PP1 to replication origins to reverse DDK-dependent MCM phosphorylation.

This is an open access article under the CC BY license (http://creativecommons.org/licenses/by/4.0/).

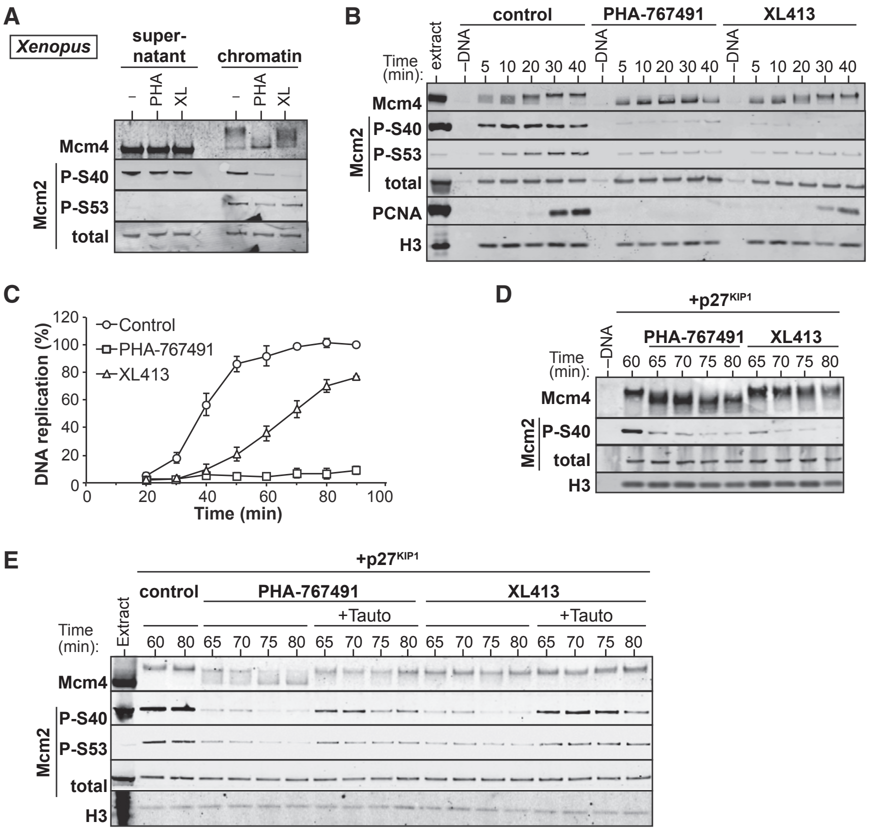

Figure 1. Phosphorylation of Different MCM Subunits by *Xenopus* DDK Is Reversed by PP1

(A–C) *Xenopus* egg extracts were supplemented with demembranated sperm nuclei and optionally supplemented with 50 μM PHA-767491 (PHA) or 100 μM XL413 (XL). (A) After incubation for 40 min, isolated chromatin along with 0.5% (v/v) of the supernatant was immunoblotted for Mcm4, Mcm2-P-S40, Mcm2-P-S53, and total Mcm2. (B) At the indicated times, chromatin was isolated and immunoblotted for Mcm4, Mcm2-P-S40, Mcm2-P-S53, total Mcm2, PCNA, and histone H3. (C) Extract was supplemented with [α-^{32}P] dATP. Total DNA synthesis was determined at indicated times.

(D and E) Sperm nuclei were incubated for 60 min in *Xenopus* egg extracts treated with p27^{kip1} to allow Mcm4 hyperphosphorylation. 50 μM PHA-767491 or 100 μM XL413 plus or minus 1 μM tautomycetin (Tauto) was then added, and chromatin was isolated either immediately after inhibitor addition (60 min) or later at every 5 for next 20 min (65–80 min). Chromatin samples were immunoblotted for Mcm4, Mcm2-P-S40, Mcm2-P-S53, total Mcm2, and histone H3.

In this paper, we show that DDK-dependent hyper-phosphorylation of Mcm4, but not phosphorylation of Mcm2, correlates well with replication initiation in both *Xenopus* extracts and human cells. We show that Rif1-directed PP1 opposes DDK-dependent phosphorylation on MCMs, acting in parallel with the ATR-dependent checkpoint pathway. In human cells, loss of Rif1 results in accelerated progression through S phase due to increased initiation, disrupting the normal replication timing program. Loss of Rif1 can also alleviate the slow progression through S phase that results when DDK is inhibited. Finally, we provide evidence that Rif1-PP1 destabilizes replication forks that have stalled in the absence of Chk1 activity.

RESULTS

DDK Differentially Phosphorylates MCM2–7 Subunits

The phosphorylation of Mcm2 on S40 and S53 has been exploited as a readout for Cdc7 kinase activity and has been used in screens for the development of small-molecule inhibitors of Cdc7 (Montagnoli et al., 2008; Koltun et al., 2012). We used *Xenopus* egg extracts to evaluate the hyper-phosphorylation of Mcm4 and the phosphorylation of Mcm2 at the *Xenopus* orthologous S40 and S53 residues (S25 and S38, respectively) as readouts for the DDK activity required for DNA replication (Figure 1A). As reported previously, DNA-bound Mcm4 was hyper-phosphorylated by DDK (Poh et al., 2014). Treatment with the DDK inhibitor PHA-767491 resulted in completely dephosphorylated Mcm4. Surprisingly, the more specific and potent DDK inhibitor XL413 (Hughes et al., 2012) caused only inefficient inhibition (Figure 1A). Phosphorylation of Mcm2 S53 behaved similarly to Mcm4, appearing only on chromatin and being reduced by DDK inhibition. In contrast, phosphorylation of S40 was strong in the chromatin fraction, as well as in the supernatant where it was insensitive to DDK inhibition.

To further investigate the effects of PHA-767491 and XL413 on different MCM phosphorylation sites, we isolated chromatin at short time intervals (Figure 1B) while monitoring DNA replication (Figure 1C). PHA-767491 efficiently inhibited phosphorylation of all assayed MCMs, including chromatin-bound Mcm2 at S40. As detailed previously (Poh et al., 2014), PHA-767491 also abolished DNA replication (Figure 1C). In contrast, XL413 only delayed the hyper-phosphorylation of Mcm4, although it efficiently inhibited Mcm2 phosphorylation on S40 and S53 (Figure 1B). XL413 delayed progression through S phase but did not prevent most of the DNA from being replicated. This shows that, among the different DDK-dependent MCMs phosphorylations, Mcm4 phosphorylation provides a better functional correlation with DNA replication than phosphorylation of Mcm2 on S40 or S53.

After DDK phosphorylates the Mcm2–7 complex, CDK activity promotes the initiation of replication (Jares and Blow, 2000; Walter, 2000). We used a CDK inhibitor, p27^{kip1}, to prevent replication and, hence, stabilize phosphorylated MCMs on DNA. We previously showed that, in these conditions, inhibition of DDK activity leads to rapid dephosphorylation of Mcm4 by PP1 (Poh et al., 2014). Inhibition of DDK with either PHA-767491 or XL413 resulted in the loss of Mcm2 S40 and S53 phosphorylation, but only PHA-767491 caused efficient dephosphorylation

of Mcm4 (Figures 1D and 1E). When tautomycetin, a specific inhibitor of PP1 (IC_{50} [half maximal inhibitory concentration] = 1.6 nM and 62 nM for PP1 and PP2, respectively), was added along with PHA-767491 or XL413, virtually all MCM dephosphorylation was abolished (Figure 1E). This suggests that PP1 activity reverses DDK-dependent phosphorylation on all its MCM substrates. This result also suggests that the inability of PHA-767491 or XL413 to inhibit Mcm2 S40 phosphorylation in solution (Figure 1A) is due to the lack of PP1 activity, which, as we have previously shown, is recruited to chromatin to promote MCM dephosphorylation (Poh et al., 2014).

We next investigated the kinetics of DDK-dependent MCM phosphorylation in HeLa cells by using mitotic shakeoff to monitor cells as they move from G1 to S and onto G2 (Figure 2A). As has been previously reported (Masai et al., 2006), phosphorylation of chromatin-bound Mcm4 (evidenced by slower migrating bands on SDS-PAGE) occurred as cells entered into and progressed through S phase (Figure 2B). Consistent with results from *Xenopus*, Mcm4 in the HeLa soluble fraction behaved differently and was typically seen as one strong, fast migrating band and one slower migrating weak band that did not vary over the cell cycle. Phosphorylation of Mcm2 S40 was most evident in the soluble fraction rather than on chromatin. Moreover, the phosphorylation of soluble Mcm2 S40 was strongest in G1, it nearly disappeared during S phase, and it returned as cells moved into G2 phase.

The efficacy of both PHA-767491 and XL413 has been assessed by their ability to inhibit Cdc7-dependent phosphorylation of soluble Mcm2 in human tissue culture cells (Montagnoli et al., 2008; Koltun et al., 2012). To our knowledge, neither was assessed for Mcm4 phosphorylation status. To test this, HeLa cells were replated after a mitotic shakeoff and then challenged with PHA-767491 or XL413. Each compound inhibited DNA replication and S-phase entry, with PHA-767491 displaying stronger activity (Figure 2C). The stronger effect of PHA-767491 is likely explained by its promiscuous activity against other kinases, including Cdk9, which is required for S-phase entry (Montagnoli et al., 2008; Hughes et al., 2012). PHA-767491 induced apoptosis in HeLa cells as cells enter S phase after mitotic shakeoff (Figure S1A). However, when the inhibitors were added just after the start of S phase, PHA-767491 no longer had a strong apoptotic effect, and both PHA-767491 and XL413 appeared to inhibit S-phase entry to a similar degree (Figures S1B and S1C). PHA-767491 or XL413 also suppressed phosphorylation of chromatin-bound Mcm4, as well as S40 and S53 of Mcm2, consistent with these being direct DDK targets (Figure 2D). Congruent with results in *Xenopus* egg extracts, inhibition of PP1 with tautomycetin resulted in the accumulation of phosphorylated, chromatin-bound Mcm2 and Mcm4. Tautomycetin also reduced the dephosphorylation of chromatin-bound MCMs induced by XL413 or PHA-767491. In contrast to the experiments in egg extracts, both inhibitors reduced phosphorylation of soluble Mcm2, suggesting more active dephosphorylation of soluble Mcm2 in human cells. Both XL413 and PHA-767491 consistently increased the proportion of cells that remained in the near-2N population (Figure S1C) when added to cells synchronized in S phase; co-addition of tautomycetin partially reversed this effect. These results show that, in both *Xenopus* egg extracts and human cells, phosphorylation of Mcm4, but not Mcm2, provides the best indicator of DDK activity required for DNA replication initiation and that phosphorylation of chromatin-bound MCMs by DDK is counteracted by phosphatase activity of PP1.

PP1 Requires Rif1 to Dephosphorylate the Mcm2–7 Complex

Previous data in yeast has established that Rif1 recruits PP1 to replication origins to dephosphorylate MCMs (Davé et al., 2014; Hiraga et al., 2014; Mattarocci et al., 2014). The motifs on Rif1 required for interaction with PP1 are conserved in *Xenopus* and mammalian Rif1 homologs but are located within the C terminus of the protein (Sreesankar et al., 2012). In order to test whether Rif1 directs PP1 to replication origins in vertebrates, we raised an antibody to *Xenopus* Rif1 (Figure S2A). Immunoprecipitation of Rif1 from *Xenopus* egg extracts co-precipitated PP1γ and PP1β (Figure 3A). Moreover, PP1β was detected by mass spectrometry in immunoprecipitates of Rif1 from chromatin (Figure S3A). Inhibition of *Xenopus* Cdc7 did not obviously reduce the Rif1-PP1 interaction (Figure S3B) as observed in yeast (Hiraga et al., 2014). In human tissue culture cells PP1α and PP1γ, Mcm2 and Cdc7 were co-precipitated with chromatin-bound Rif1 (Figure 3B).

If Rif1 targets PP1 to DNA-bound Mcm2-7, then depleting Rif1 from *Xenopus* egg extracts should mimic the effect of inhibiting PP1. Depletion of >90% of Rif1 from *Xenopus* egg extracts (Figure S3C) substantially decreased the amount of PP1 on chromatin and reduced the rate of dephosphorylation of Mcm4 and Mcm2 that occurred after DDK inhibition (Figures 3C and S3D). A more robust Rif1 depletion (>95%) caused even greater loss of chromatin bound Rif1 and PP1γ with a corresponding increase in Mcm4 phosphorylation (Figures S3E and S3F). The process of immunodepletion slightly compromises Cdc7 nuclear import (Poh et al., 2014), reducing Mcm4 hyper-phosphorylation (Figures 3C, S3D, and S3F) and sensitizing DNA replication to further DDK inhibition (Figure S4A).

We performed parallel experiments in asynchronous HeLa cells using small interfering (si)RNA against Rif1. Hyper-phosphorylated Mcm4 accumulated in proportion to the amount of Rif1 knocked down by RNAi treatment (Figure 3D). The accumulation of hyper-phosphorylated Mcm4 was more pronounced in cells synchronized in S phase following a mitotic shakeoff (Figure 3E). As we observed in *Xenopus* (Figure 3C), knockdown of Rif1 in human cells rendered phosphorylation of Mcm4 relatively insensitive to DDK inhibition (Figure 3E). These data clearly show that, in both *Xenopus* and human systems, loss of Rif1 increases DDK-dependent phosphorylation of MCMs in a manner that is similar to inhibition of PP1 and implicates Rif1 in directing PP1 to origins of replication.

Rif1 Is Essential for Regulating S-phase Progression

We next investigated the potential role of Rif1 in regulating progression into and through S phase. When *Xenopus* extracts were immunodepleted of Rif1, the rate of replication increased compared to control immunoglobulin-G (IgG)-depleted extract (Figures 4A and S4B). Furthermore, Rif1 depletion reduced the inhibitory effect of PHA-767491 on DNA replication (Figure 4B).

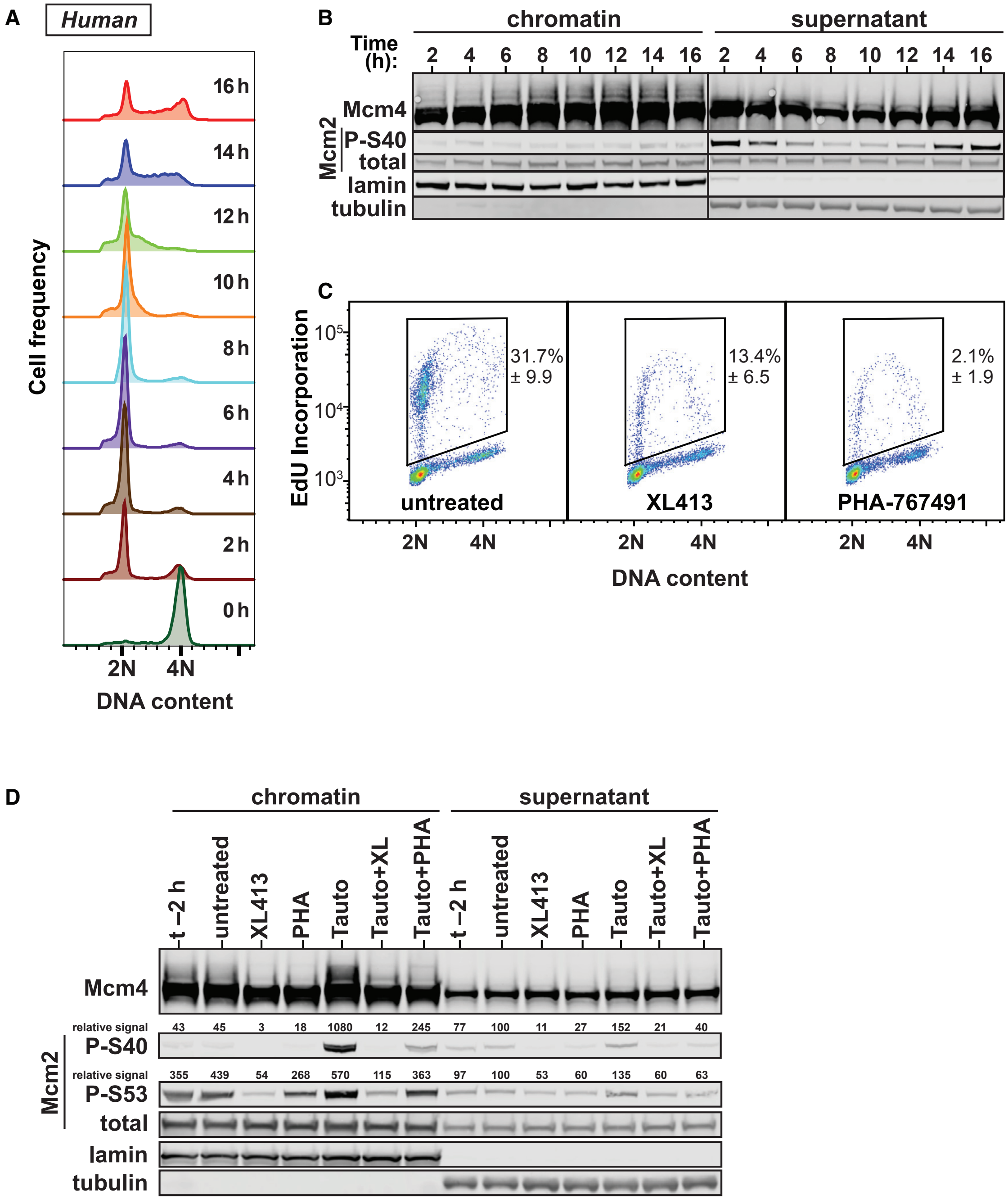

Figure 2. Human Mcm4 Phosphorylation Predicts DNA Replication

(A and B) HeLa cells were synchronized in early M phase by mitotic shakeoff and then replated for different times. (A) DNA content of cells as determined by flow cytometry. (B) Immunoblot of Mcm4, total Mcm2, and Mcm2-P-S40 on and off chromatin. Lamin B and tubulin serve as loading and fractionation controls.

(C) Cells were synchronized as in (A) and were replated with 10 μM PHA-767491 or 10 μM XL413 in the media. At 7.5 hr, cells were pulsed for 30 min with EdU and then analyzed by flow cytometry for DNA content and EdU incorporation. An example plot and gate are shown plus the mean and SD of EdU+ cells from three experiments.

(D) Cells were synchronized as in (A) and replated for 8 hr (t-2); media was then optionally supplemented with 10 μM PHA-767491 (PHA), 10 μM XL413 or 225 nM tautomycetin (Tauto) as indicated for 2 hr. Cells were then immunoblotted for Mcm4, Mcm2-P-S40, Mcm2-P-S53, or total Mcm2 on and off chromatin. Lamin B and tubulin served as loading and fractionation controls.

See also Figure S1.

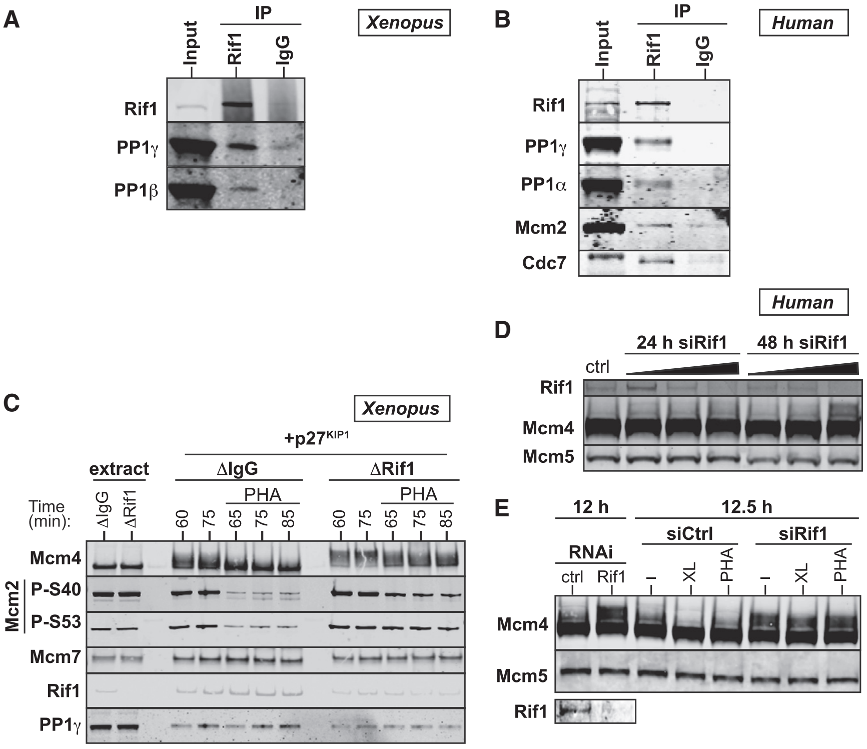

Figure 3. Rif1 Targets PP1 to MCMs at Replication Origins

(A and B) Rif1 was immunoprecipitated from *Xenopus* egg extract (A) or HeLa cell nuclear extracts (B) using antibodies against Rif1 or with pre-immune rabbit IgG and samples were immunoblotted for Rif1, PP1γ, PP1β, PP1α, Mcm2 or Cdc7 as indicated.

(C) Sperm nuclei were incubated for 60 min in *Xenopus* extracts immunodepleted using antibodies against Rif1 (ΔRif1) or control pre-immune rabbit IgGs (ΔIgG). Sperm nuclei and $p27^{kip1}$ were added to extracts to allow Mcm4 hyper-phosphorylation. Chromatin was isolated at 60 and 75 min post-incubation. Optionally, after 60 min of incubation with DNA, 50 μM PHA-767491 (PHA) was added, and chromatin was isolated at indicated times over the next 25 min (65–85 min). Chromatin samples were immunoblotted for Mcm4, Mcm2-P-S40, Mcm2-P-S53, Mcm7, Rif1, and PP1γ.

(D) HeLa cells were transfected with increasing amounts of siRNA targeting Rif1 and tested for knockdown efficiency as well as Mcm4 phosphorylation at 24 and 48 hr after transfection. Mcm5 served as a loading control (Ctrl).

(E) ~48 hr after transfection with Rif1 or control RNAi, HeLa cells were synchronized in S phase by mitotic shakeoff and treated for 30 min with either 10 μM PHA-767491 (PHA) or 10 μM XL413 (XL). Chromatin-bound Mcm4 was analyzed by immunoblotting, with Mcm5 serving as a loading control. See also Figure S2.

These results show that, in *Xenopus* egg extracts, Rif1 counteracts the function of DDK in driving S-phase progression.

A similar effect was seen when Rif1 levels were reduced in human cells using RNAi. On release from a double thymidine block, siRif1 HeLa cells moved more quickly through S phase and incorporated 5-ethynyl-2′-deoxyuridine (EdU) at a higher rate than control cells (Figures 4C–4E). Moreover, the inhibition of S-phase progression observed with XL413 treatment was greatly decreased in siRif1 cells. This is consistent with the idea that cells move through S phase at an enhanced rate when DDK activity is not restrained by Rif1-PP1.

The enhanced rate of S phase progression observed in cells deficient for Rif1 could, in principle, be due to increased replication initiation and/or faster replication fork movement. To test this, we assessed progression through the replication timing program by observing the spatial pattern of DNA synthesis in cells released from a double thymidine block (O'Keefe et al., 1992; Gillespie and Blow, 2010). Figure 5A shows that EdU incorporation in Rif1-depleted cells was abnormal, both in its intensity and in showing less discrete labeling patterns. Incorporation of EdU 90 min after release from a thymidine block was greater in the siRif1 cells in comparison to the siCtrl cells (Figure 5B), in agreement with their faster progression through S phase (Figure 4E). The number of replication foci per nucleus was slightly lower in the siRif1-treated cells (Figure 5C), though the intensity of the observed EdU foci nearly doubled (Figure 5D). This could represent the activation of more replication domains that partially overlap with one another, leading to an apparent decrease in the number of foci. At later time points after thymidine release, there were no major differences between siCtrl and siRif1-treated cells (Figure S5), implying that aberrant EdU incorporation due to the absence of Rif1 occurs primarily in early S phase.

DNA fiber analysis showed that the replication fork speed was similar in siCtrl and siRif1-treated cells (Figure 5E). Previous work in mammalian cells has shown that loss of Rif1 results in limited change in inter-origin distances (Cornacchia et al., 2012) and a highly disrupted chromatin structure (Foti et al., 2016). Therefore, the increased levels of EdU incorporation we observed after Rif1 depletion likely reflects initiation occurring across larger timing domains at an otherwise unchanged origin density, consistent with an underlying change in chromatin structure.

Rif1 Regulates S-phase Progression in Parallel with ATR/Chk1

Previous work has implicated Rif1 in the regulation of replication initiation in response to activation of cell-cycle checkpoints (Kumar and Cheok, 2014; Renard-Guillet et al., 2014). Therefore, we sought to understand whether Rif1 was regulated by the S-phase checkpoint kinases ATM and ATR. We observed S phase-progression of synchronized HeLa cells after treatment with an ATM inhibitor (KU55933), a Chk1 inhibitor (CHIR-124), or a generic inhibitor of ATM/ATR/DNA-PK (caffeine) in the presence and absence of Rif1 (Figure 6). Both caffeine and CHIR-124, but not KU55933, inhibited S-phase progression in siCtrl cells, consistent with the known role of ATR and Chk1 in promoting fork stability (Smits and Gillespie, 2015). siRif1 cells treated with caffeine or CHIR-124 showed an increase in EdU incorporation and S-phase progression (Figure 6A). This implies that Rif1 has a negative

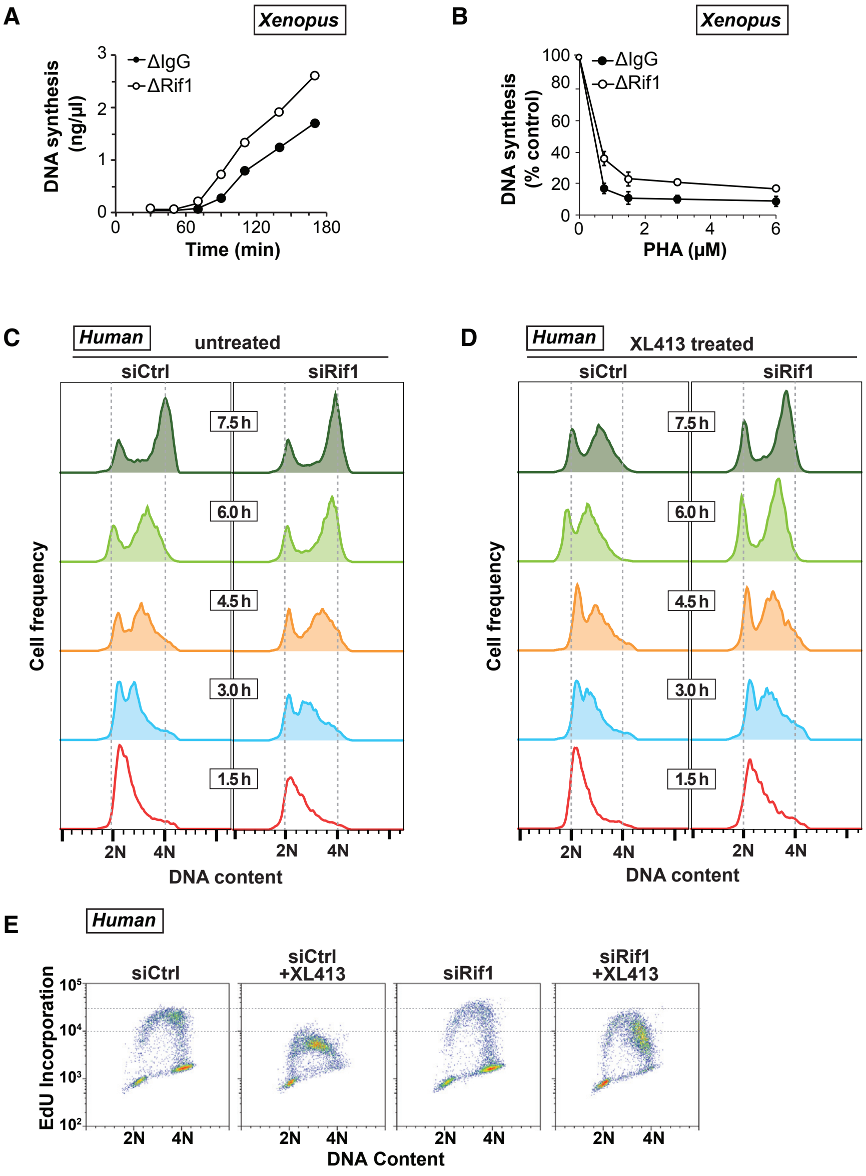

Figure 4. Rif1/PP1 Activity Is Required to Oppose Cdc7 Activity for Normal Replication

(A and B) Control (ΔIgG) and Rif1 (ΔRif1)-depleted *Xenopus* extracts were incubated with demembranated sperm nuclei and [α-^{32}P]dATP. (B) Extracts were optionally supplemented with increasing concentrations of PHA-767491 (PHA) (0.75–6 µM). The reactions were stopped at the indicated times (A) or at 150 min (B), and total DNA synthesis was determined.

(C and D) Flow cytometry was used to analyze cellular DNA content in the following experiment: HeLa cells were transfected with control or Rif1 siRNA, synchronized by a double thymidine block, and released into untreated (C) or XL413-treated (D) media for the indicated times.

(E) Cells from the 6-hr time points from (C) and (D) were pulsed with EdU and analyzed by flow cytometry.

See also Figure S4.

regulatory effect on S-phase progression that is independent of ATR and Chk1. To determine whether this Rif1 function occurs by opposing DDK activity, we observed S-phase progression in synchronized cells treated with a DDK inhibitor, a Chk1 inhibitor, or both in the presence and absence of Rif1. In control cells, both CHIR-124 and XL413 individually caused a slight reduction in EdU incorporation, which was enhanced when combined with each other (Figure 6B). In contrast, XL413 had no significant effect on EdU incorporation or S-phase progression in siRif1 cells, either alone or when combined with CHIR-124. This strongly implies that, in the absence of exogenously applied replicative stresses, Rif1 restricts S-phase progression by opposing DDK function in a manner that is independent of ATR and Chk1.

We next investigated Rif1 function under conditions of replication stress. It has previously been shown in *Xenopus* egg extracts that the topoisomerase II inhibitor etoposide inhibits DDK activity via both checkpoint-dependent and checkpoint-independent pathways (Costanzo et al., 2003; Poh et al., 2014). Therefore, we added etoposide to control and Rif1-depleted HeLa cells synchronized in early S phase. As expected, this caused a reduction in EdU incorporation and S phase progression (Figure 6C). When treated with both etoposide and CHIR-124, control cells had great difficulty proceeding with S phase, and incorporation of EdU was significantly decreased. This is consistent with the known role of Chk1 in stabilizing stalled replication forks, as confirmed by DNA fiber analysis, which showed a decrease in the average labeled track length of ~50% (Figure 6D). Remarkably, in Rif1-deficient cells, co-treatment of etoposide and CHIR-124 resulted in near-normal replication profiles as judged by fluorescence-activated cell sorting (FACS). DNA fiber analysis showed that, in Rif1-deficient cells, the length of labeled tracks was largely unaffected by etoposide or CHIR-124. These data are consistent with the idea that, in addition to its role in regulating initiation, Rif1 destabilizes replication forks that have stalled in the absence of Chk1 activity.

PP1-Dependent Dephosphorylation of CMG Destabilizes Replication Forks

The results presented earlier show that Rif1-PP1 reverses DDK-dependent phosphorylation of chromatin-bound MCMs. However, at any given moment, only a fraction of the total chromatin-bound MCMs are active as replicative CMG helicases. Therefore, we analyzed the effect of Rif1-PP1 on CMG complexes using *Xenopus* egg extracts. DNA was incubated for 60 min in extract containing 100 µM aphidicolin (to inhibit

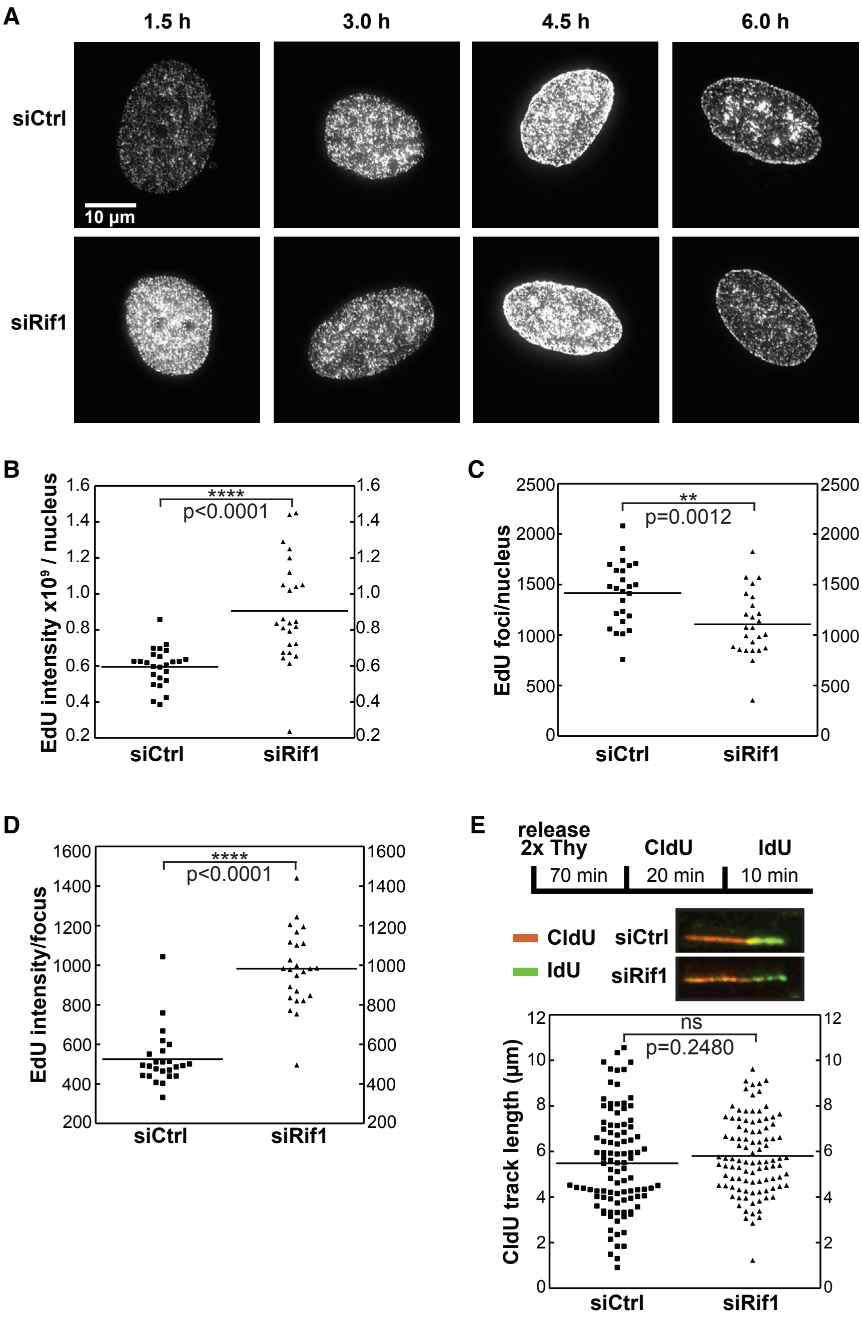

Figure 5. Rif1 Restrains Replication Initiation

(A–D) HeLa cells were treated with control or Rif1 siRNA, synchronized by double thymidine block, and then released. (A) At the indicated periods after release, cells were pulsed with EdU for 30 min. The spatial patterns of DNA synthesis were examined by fluorescence microscopy. (B–D) 90 min after release, the total EdU intensity of the nucleus (B), the number of replication foci per nucleus (C), and the intensity of the EdU foci (D) were quantified (25 nuclei per condition).

(E) Cells were labeled for 20 min with CldU 70 min after release from thymidine (Thy), washed, and pulsed briefly with IdU (10 min). DNA fibers were spread, and the length of 100 CldU-labeled tracks was measured. Data were evaluated for significance using an unpaired, two-tailed t test.

See also Figure S5.

replication fork progression) and 5 mM caffeine (to allow dormant origins to fire, thereby increasing the number of active CMGs) (Woodward et al., 2006). PHA-767491 and/or tautomycetin were then added, after which CMG complexes were isolated by immunoprecipitation of Cdc45 (Figure 7A). DDK inhibition caused significant dephosphorylation of total chromatin-bound MCM4 but only partial dephosphorylation of MCM4 in the CMGs. In both cases, dephosphorylation was inhibited by tautomycetin. However, there was strong PP1-dependent dephosphorylation of MCM2 S40, both on total chromatin-bound MCM2 and in the CMGs. MCM2 S53 phosphorylation was not significantly affected by DDK inhibition. These results suggest that PP1-dependent MCM dephosphorylation occurs on the CMG, but with different kinetics at different MCM sites compared to MCM complexes at licensed origins.

Previous work has shown that, unlike the case in mammalian cells, replisomes in *Xenopus* extracts are not destabilized when Chk1 is inhibited (Luciani et al., 2004). Since our results in human cells indicate that replisome stability involves both MCM phosphorylation and Chk1 activity (Figure 6D), we considered whether the very high degree of DDK-mediated MCM phosphorylation in *Xenopus* egg extracts (Figure 1) helps stabilize replisomes, even when Chk1 is inhibited. To test this, replication forks in *Xenopus* egg extracts were stalled with aphidicolin, new initiation events were blocked with p27^{kip1}, and the incubation was continued in the presence of PHA-767491 plus or minus caffeine (which, by inhibiting ATR, prevents Chk1 activation). Chromatin was then isolated, and replication fork elongation was measured in a run-on assay (Figure 7B). When caffeine was added in addition to PHA-767491, the overall rate of replication was reduced to 76% ± 5% (mean ± SEM, six independent experiments; $p < 0.05$, an unpaired, two-tailed t test). The results of the experiments shown in Figures 7A and 7B are consistent with the data from HeLa cells in Figure 6D, and they suggest that, in the absence of ATR/Chk1 activity, DDK-dependent phosphorylation of CMG complexes becomes important to maintain replisome stability.

Finally, we investigated Rif1's ability to inhibit S-phase progression in response to a range of different replication stresses. To this end, we observed EdU incorporation in the presence and

OPEN
ACCESS
CellPress

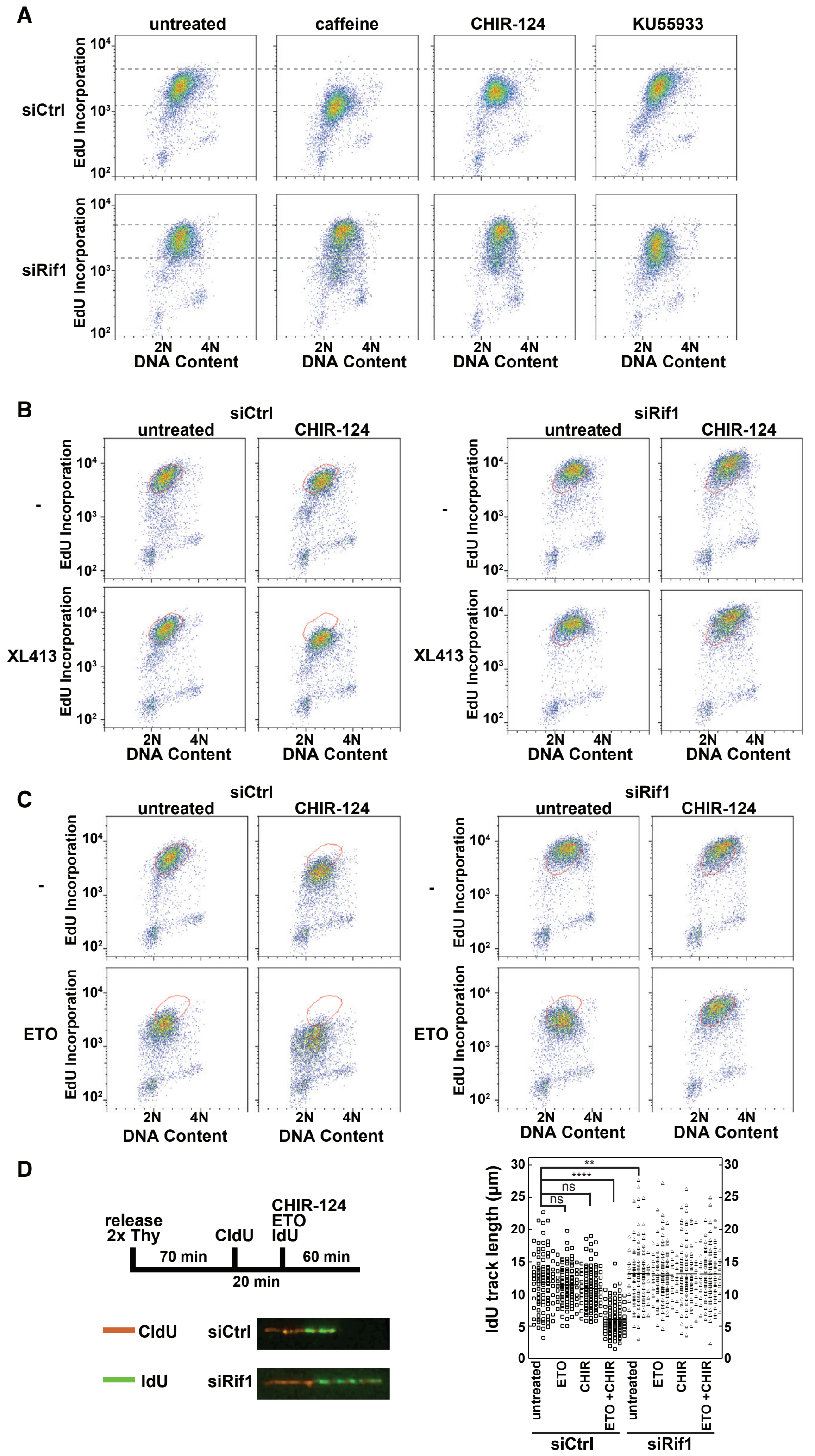

Figure 6. Rif1 Regulates S-phase Progression and Replisome Stability in Parallel with ATR/Chk1

(A–C) HeLa cells were treated with control or Rif1 siRNA, synchronized and released from a double thymidine block for 2 hr, and then optionally treated for 2 hr with caffeine (5 mM), CHIR-124, KU55933, XL413, or etoposide as indicated. EdU was added in the last 30 min of treatment and the cells were analyzed by flow cytometry.

(D) HeLa cells were treated with control or Rif1 siRNA, synchronized and released from a double thymidine (Thy) block for 70 min, labeled with CldU for 20 min, washed, and subsequently labeled with IdU while being incubated with CHIR-124 (CHIR), etoposide (ETO), or both for 1 hr. DNA fibers were prepared, and the IdU length of 100 tracks were counted per experimental condition. Data were evaluated for significance using an unpaired, two-tailed t test, with significant differences in fiber length between siCtrl untreated and siCtrl ETO + CHIR ($p < 0.0001$), and between siCtrl untreated and siRif1 untreated ($p = 0.0321$). There was no significant difference found between any siRif1 treatments.

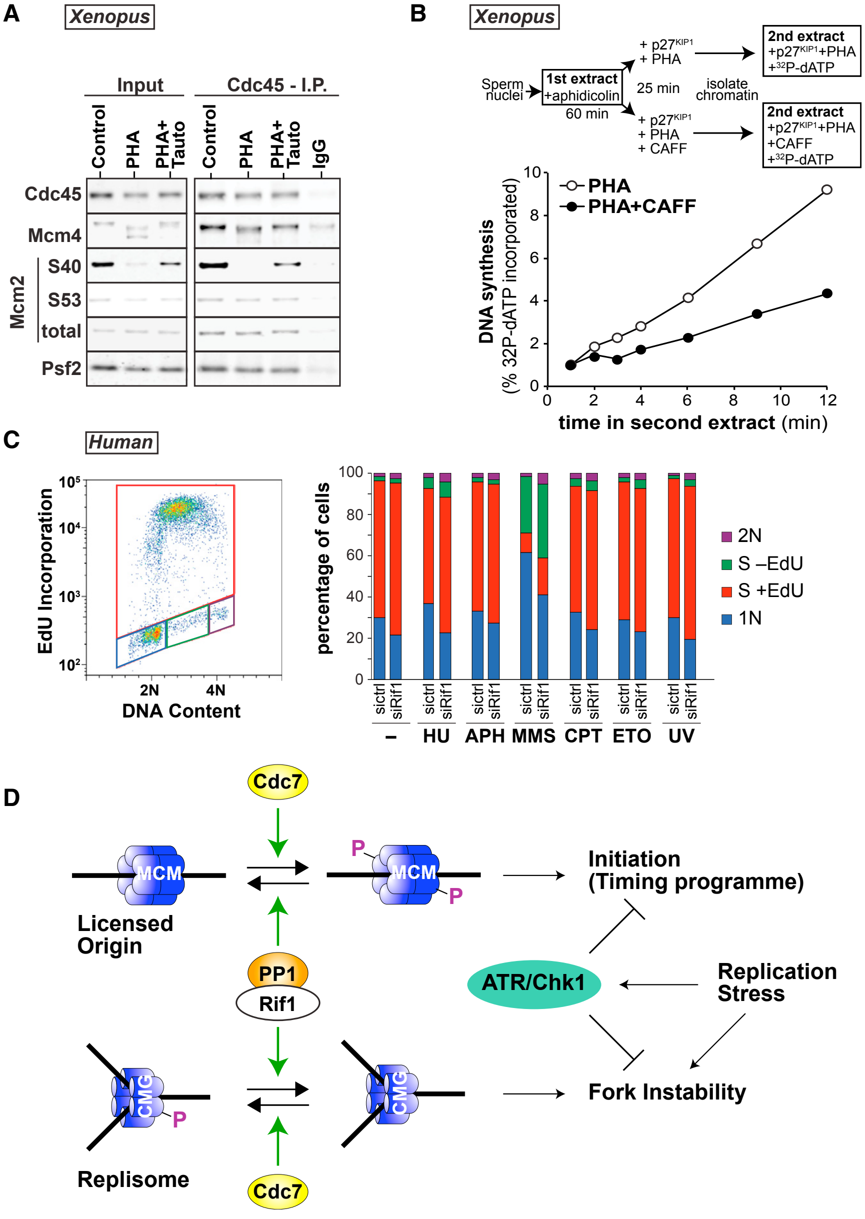

Figure 7. Rif1-PP1 Regulates Replisome Stability and S-phase Progression

(A) Sperm nuclei were incubated for 60 min in *Xenopus* egg extracts supplemented with 100 μM aphidicolin and 5 mM caffeine. 50 μM PHA-767491 (PHA) ± 1 μM tautomycetin (Tauto) was added for a further 25 min. Chromatin-bound proteins were immunoprecipitated with antibodies against Cdc45 or pre-immune sheep IgG. Samples were immunoblotted for Cdc45, Mcm4, Mcm2-P-S40, Mcm2-P-S53, total Mcm2, and Psf2.

(B) *Xenopus* egg extracts were supplemented with demembranated sperm nuclei and 100 μM aphidicolin. After 60 min, $p27^{kip1}$ was added and aliquots were supplemented with 50 μM PHA-767491 ± 5 mM caffeine (CAFF). After a further 25 min, chromatin was transferred to extract supplemented with [α-^{32}P]dATP, p27kip1 and 50 μM PHA-767491 ± 5 mM caffeine to match the first incubation. At the indicated times, incorporation of [α-^{32}P]dATP into nascent DNA was determined and expressed as normalized values against the incorporation at 1 min.

(C) HeLa cells were treated with control or Rif1 siRNA, synchronized by double thymidine block, and released 2 hr before either treatment with hydroxyurea (HU; 5 mM), aphidicolin (APH; 1 μg/mL), methyl methanesulfonate (MMS; 0.02%), camptothecin (CPT; 5 μM), etoposide (ETO; 5 μM), or UV (~45 J/m^2). EdU was added in the last 30 min of treatment, and 2 hr after the start of the treatments, cells were harvested, fixed, and analyzed by flow cytometry.

(D) Model for the role of Rif1 and Cdc7 in controlling replication initiation and replisome stability. Replication initiation is regulated by the phosphorylation of Mcm2-7 by Cdc7, opposed by Rif1/PP1. Active replisomes are also dephosphorylated by Rif1/PP1, which requires ATR/Chk1 to stabilize them in response to replicative stresses.

See also Figure S6.

absence of Rif1 in HeLa cells exposed to a variety of drugs: 5 mM hydroxyurea, 1 μg/mL aphidicolin, 0.02% methyl methanesulfonate, 5 μM camptothecin, and 5 μM etoposide (or 45 J/m^2 UV light) (Figures 7C and S6). In each case, control cells responded by reducing the rate of S-phase entry and decreasing the population of cells incorporating EdU. However, in Rif1 knockdown cells, the response to all these inhibitors was diminished, leading to a decrease in the near-2N population and a corresponding increase in cells incorporating EdU.

DISCUSSION

We show here how the interplay between DDK-dependent phosphorylation and Rif1-PP1-dependent dephosphorylation of MCM proteins regulates S-phase progression and replisome stability. DDK-dependent hyper-phosphorylation of Mcm4, but not Mcm2, correlates well with replication initiation in both *Xenopus* and humans. Rif1-directed PP1 dephosphorylates DDK-dependent phosphorylation on all MCMs. Loss of Rif1 accelerates progression through the S phase due to increased initiation, disrupting the normal replication timing program independently of the ATR-dependent checkpoint pathway. We also show that Rif1 is required to destabilize replication forks that have stalled in the absence of ATR-Chk1 activity.

MCM4 Phosphorylation Correlates with Replication Initiation

We have shown that DDK-dependent phosphorylation sites on different MCMs are differentially regulated, with slight variations between *Xenopus* and human systems. Mcm4 hyper-phosphorylation, but not MCM2 phosphorylation, at S40 and S53

correlates well with replication initiation. This is in agreement with studies in yeast where phosphorylation of the N terminus of Mcm4 is required to relieve an inhibitory function on replication initiation (Sheu and Stillman, 2010).

Unexpectedly, we found that XL413 is a poor inhibitor of replication in comparison to PHA-767491 in *Xenopus* egg extracts. This could be explained by XL413's poor inhibition of DDK activity on Mcm4. In vitro, XL413 is much more specific and has more extensive van der Waals interactions within Cdc7's active site than PHA-767491 (Hughes et al., 2012). However, extended XL413 treatment in HeLa cells seems to merely delay progression through S phase rather than completely shut down replication. This perhaps provides a mechanistic explanation for why XL413 fails to induce apoptosis efficiently in cancer cells (Sasi et al., 2014). It is also important to note that several drug screens have been carried out using Mcm2 S40 phosphorylation as a marker of DDK activity. This phosphorylation can be observed off chromatin and outside of S phase, and it is possible that there is a structurally different form of DDK that phosphorylates MCMs off chromatin.

Rif1 Controls S-phase Progression and Replisome Stability

We have established that vertebrate Rif1 interacts with PP1. We show that, consistent with data from yeast (Davé et al., 2014; Hiraga et al., 2014; Mattarocci et al., 2014), Rif1 promotes the PP1-mediated dephosphorylation of MCMs, both in humans and *Xenopus*. We show that, in both these systems, one of Rif1's functions is to restrain replication initiation by reversing DDK-mediated phosphorylation of MCMs. Previous work has shown that loss of Rif 1 leads to an aberrant replication timing phenotype of Rif1-deficient cells (Cornacchia et al., 2012; Hayano et al., 2012; Yamazaki et al., 2012; Foti et al., 2016). Our data are consistent with the idea that loss of the phosphatase causes premature Mcm4 hyper-phosphorylation, thus causing promiscuous activation of replication origins and disruption of the timing program (Figure 7D, top). This function of Rif1 can occur concomitantly with its proposed role in organizing 3D chromatin structure, which was recently shown to affect replication timing and 3D nuclear organization in mouse cells (Foti et al., 2016). This altered 3D chromatin structure may cause improper regulation of MCM phosphorylation.

Significantly, combining the loss of Rif1 with inhibition of ATR/Chk1 enhances the already increased rate of EdU incorporation and progression through S phase seen in cells deficient in Rif1 alone. This is consistent with the idea that DDK/Rif1 and ATR/Chk1 provide distinct restraints on replication initiation, with DDK/Rif1 regulating MCM phosphorylation and ATR/Chk1 regulating CDK activity.

We also show that there is a PP1-dependent dephosphorylation of MCMs in active CMG helicases, with a different spectrum of MCM dephosphorylation than that seen on unfired MCM2-7 hexamers. When replication fork progression is inhibited, ATR/Chk1 becomes essential to prevent irreversible fork stalling (Smits and Gillespie, 2015; Sørensen et al., 2005). Our data suggest that the fork stability function of ATR/Chk1 is critical because of the action of Rif1. In *Xenopus*, extract replication forks, which are normally relatively insensitive to ATR/Chk1 inhibition (Luciani et al., 2004), become unstable when combined with DDK inhibition. In HeLa cells, inhibition of Rif1-PP1 prevents the inactivation of replication forks that occurs when the ATR/Chk1 pathway is inhibited. The simplest explanation for this fork stability function of Rif1 is that it is associated with Rif1's ability to drive MCM dephosphorylation, though it is also possible that replisome stability depends on the phosphorylation of other replisome proteins.

In this model (Figure 7D, bottom), DDK-dependent MCM phosphorylation of the active CMG helicase helps maintain it in an active conformation. Only once dephosphorylated by Rif1-PP1 does the replisome become susceptible to destabilization, and then the fork stability function of ATR/Chk1 becomes particularly important. This second role of Rif1 has interesting implications for the use of DDK inhibitors as anti-cancer agents. Since replisome stalling is likely to be particularly difficult to reverse, combining replication stresses with DDK inhibitors is likely to prove significantly more toxic to cancer cells lacking proper checkpoint controls.

EXPERIMENTAL PROCEDURES

Xenopus Egg Extract and DNA Templates

Metaphase-arrested *Xenopus laevis* egg extract and demembranated *Xenopus* sperm nuclei were prepared and used as described previously (Gillespie et al., 2016). Briefly, extracts were released into interphase from metaphase arrest once incubated with 0.3 mM $CaCl_2$ for 15 min at 23°C. For DNA synthesis reactions, sperm nuclei were incubated at 3–10 ng DNA/μL in extract. DNA synthesis was assayed by measuring incorporation of [α-^{32}P] dATP into acid-insoluble material followed by scintillation counting (Gillespie et al., 2016).

Chromatin Isolation from Egg Extract

Chromatin isolation from egg extract was carried out as described previously (Gillespie et al., 2012). Briefly, extract was diluted with ice-cold nuclear isolation buffer (NIB) (50 mM KCl, 50 mM HEPES-KOH [pH 7.6], 5 mM $MgCl_2$, 0.5 mM spermidine, 0.15 mM spermine, and 2 mM DTT) containing phosphatase inhibitors, under-laid with 20% sucrose (w/v) in NIB, and centrifuged in a swinging-bucket rotor at 2,100 × *g* for 5 min at 4°C. Following a cushion wash, chromatin was spun down at 13,000 × *g* for 2 min in a fixed-angle rotor. The resulting pellet was resuspended in SDS loading buffer.

To isolate intact nuclei for transfer experiments, Triton X-100 was omitted from all buffers. Extracts were diluted as before, under-laid with a double cushion of NIB + 20% sucrose and NIB + 30% glycerol (v:v in NIB), and centrifuged in a swinging bucket rotor. Following a cushion wash, nuclei were resuspended in the glycerol cushion and added to the second extract at a final DNA concentration of 10 ng/μL.

Cell Culture and Synchronization

HeLa cells were obtained from the American Type Culture Collection (ATCC) and maintained in DMEM (Invitrogen) supplemented with 10% fetal bovine serum (FBS; Invitrogen) and antibiotics at 37°C in 5% CO_2. For mitotic shake-off experiments, HeLa cells were synchronized in early S phase via double thymidine block, washed with PBS, and released into media for 6 hr before treatment with nocodazole at 100 ng/mL for 4 hr. Flasks were knocked onto the benchtop to release mitotic cells and then were washed with pre-warmed PBS and replated.

Protein Preparation and Isolation of Chromatin Proteins in Human Cells

Whole-cell extracts were obtained from HeLa cells by extraction in RIPA buffer (50 mM Tris-HCl [pH 7.4], 150 mM NaCl, 1% IGEPAL CA-630, 0.5% sodium deoxycholate, 1 mM EDTA, 1 mM PMSF, 1 mM sodium orthovanadate,

PhosSTOP (04906845001, Roche). For chromatin fractionation, cells were harvested from plates, washed with PBS, and resuspended in cytoskeleton (CSK) buffer (10 mM HEPES [pH 7.4]; 300 mM sucrose; 100 mM NaCl; 3 mM $MgCl_2$; 0.5% Triton X-100; 1 mM PMSF; 0.1 mM sodium orthovanadate; PhosSTOP [04906845001, Roche]; and 1 μg/mL each of leupeptin, aprotinin, and pepstatin) for 15 min on ice. Tubes were centrifuged at 5,000 × *g* for 5 min at 4°C, and the non-chromatin bound supernatant was frozen in liquid nitrogen. The chromatin pellet was washed twice using CSK buffer without Triton X-100, centrifuged at 5,000 × *g* for 5 min at 4°C, and frozen in liquid nitrogen. Protein was prepared from chromatin pellets by extraction in RIPA buffer.

FACS Analysis

At 30 min before collecting cell samples, EdU was added to cells (final concentration, 40 μM), after which cells were harvested and fixed with ice-cold 70% ethanol. EdU detection was achieved using the Click-IT Plus EdU Alexa Fluor 647 Flow Cytometry Assay Kit (C10635, Life Technologies), and DNA content was measured with propidium iodine. EdU incorporation and DNA content were measured using BD FACSCanto, and data were analyzed using the FlowJo program.

Immunoblotting

For immunoblotting, samples were separated on either 3%–8% Tris-acetate or 4%–12% Bis-Tris gradient gels (Invitrogen). Proteins were transferred onto PVDF (GE Healthcare, RPN303F) or nitrocellulose membranes (GE Healthcare, 10600001) using a wet transfer system, blocked in PBS with 0.2% Tween-20 and 5% non-fat milk. After incubation with primary and secondary antibodies, either membranes were developed using enhanced chemiluminescence detection (SuperSignal West Pico Chemiluminescent; Thermo Scientific, 34087), or signals were acquired using Odyssey bio-systems (LI-COR Biosciences) where LI-COR secondary antibodies were used.

Immunodepletion and Immunoprecipitation

To deplete Rif1 from *Xenopus* egg extract, metaphase-arrested extract was activated to release it into interphase, and subsequently, 1 vol of extract was incubated twice with 0.6 vol of protein A agarose beads conjugated to anti-Rif1 antibody for 45 min at 4°C.

To precipitate Rif1 from *Xenopus* egg extract, metaphase-arrested extract was released into interphase and then diluted 5-fold in LFB (licensing factor buffer) 1/50 (40 mM HEPES-KOH [pH 8.0]; 20 mM $K_2H(PO_4)/KH_2(PO_4)$ [pH 8.0]; 2 mM $MgCl_2$; 1 mM EGTA; 2 mM DTT; 10% [w/v] sucrose; 1 μg/mL each of leupeptin, pepstatin, and aprotinin; supplemented with 50 mM KCl). The diluted extract was incubated for 1 hr at 4°C with protein A agarose beads covalently conjugated to anti-Rif1 antibody with bis(sulfosuccinimidyl)suberate (21580, ThermoFisher Scientific). The beads were then washed five times with filtered PBS before resuspension in loading buffer. Cdc45 was immunoprecipitated according to Gambus et al. (2011), with modifications. Briefly, chromatin samples were isolated in 10 vol of ice-cold NIB containing 100 mM KCl and were underlaid with NIB buffer containing 30% sucrose and spun for 10 min at 2,500 × *g* at 4°C. PHA-767491 or tautomycetin were added optionally to the buffers. Proteins were released after DNA digestion with 2 U/μL Benzonase for 10 min at room temperature. After resuspension, KCl was adjusted to 100 mM, and chromatin was incubated for 5 min at room temperature before the final spin. Cdc45 antibody was covalently conjugated to Dynabeads Protein G (10003D, Thermo Fisher Scientific) with bis(sulfosuccinimidyl)suberate for 2 hr at 4°C.

Chromatin-bound Rif1 was immunoprecipitated from HeLa cells according to a procedure modified from Foti et al. (2016). HeLa cells were washed twice in ice-cold PBS before adding 5 mM bis(sulfosuccinimidyl)suberate to cross-link surface protein and were incubated on ice for 30 min. The reaction was quenched with 20 mM Tris-HCl (pH 7.5) for 15 min. Cells were washed twice with PBS and lysed in hypotonic buffer (25 mM Tris [pH 7.4], 2 mM $MgCl_2$, 50 mM KCl, and 1 mM EDTA) plus protease inhibitor cocktail (Roche) on ice for 20 min. Nuclei were washed twice in hypotonic buffer and then resuspended in RIPA buffer plus inhibitor cocktail and incubated at room temperature for 30 min with 50 U/mL benzonase. After clarification by centrifugation, lysates were subjected to co-immunoprecipitation (co-IP) using anti-Rif1 antibody covalently conjugated to Dynabeads Protein A (10008D, Thermo Fisher Scientific) with bis(sulfosuccinimidyl)suberate for 1 hr at 4°C. Beads were then washed (five times) with filtered PBS before resuspension in SDS loading buffer.

RNAi and Transfections

The following siRNA duplexes were used: siCtrl 5′-UAGCGACUAAACACAUCAA-3′ (#D0012100120, Fisher Scientific) and siRif1 (sc-62944, Santa Cruz Biotechnology). Cells were transfected using RNAiMax (#13778150, Invitrogen). Briefly, siRNA was mixed with Lipofectamine in Opti-MEM medium (#31985070, Invitrogen) and added to ~50% confluent cells grown in DMEM without antibiotics. Cells were transfected ~48 hr before each experiment.

RIPA Extraction

Cells were washed with PBS and incubated on ice for 5 min in RIPA buffer plus 1 μg/mL each of leupeptin, aprotinin, and pepstatin. Tubes were briefly sonicated and then centrifuged at ~21,000 × *g* for 10 min at 4°C. Supernatants were quantified by Bradford assay before LDS (lithium dodecyl sulfate) sample buffer (NP0007, Invitrogen) and DTT were added to a final concentration of 1×, and 50 mM, respectively. Samples were heated to 70°C for 5 min, centrifuged at maximum speed for 5 min, and loaded onto SDS-PAGE gels.

Drug and Replication Stress Treatments

All experiments with HeLa cells used drugs at the following final concentrations: XL413 (10 μM), PHA-767491 (10 μM), caffeine (5 mM), CHIR-124 (5 μM), tautomycetin (225 nM), hydroxyurea (5 mM), aphidicolin (1 μg/mL), methyl methanesulfonate (0.02%), camptothecin (5 μM), and etoposide (5 μM). UV damage was induced by removing media from the tissue culture plate and irradiating (~45 J/m^2). Media were added back, and cells were incubated for ~2 hr before harvesting.

DNA Fiber Analysis

Cells were treated with 20 μM chlorodeoxyuridine (CldU) and 100 μM iododeoxyuridine (IdU) for the indicated times, harvested, counted, and resuspended with an equal number of unlabeled cells at a concentration of 1 × 10^6 total cells per milliliter. ~2,000 cells (2-μL cell suspension) were pipetted onto a glass slide and incubated in a humid chamber for 4 min. Cells were lysed with 8 μL digestion buffer (200 mM Tris [pH 7.4], 50 mM EDTA, 0.5% SDS), incubated for 7 min in a humid chamber, and then tilted 15° to allow the DNA to spread down the length of the slide. CldU was detected with BioRad OBT0030CX antibody, and IdU was detected with BD 357580 antibody. Images were acquired using a charge-coupled device (CCD) camera connected to an Olympus IX70 deltavision deconvolution microscope under a 40× oil immersion objective. >25 fields were captured for each experiment, and 100 fibers per condition were measured using Volocity 3D Image Analysis software (PerkinElmer).

Microscopy

Cells were seeded into 10-cm dishes containing glass coverslips. Under indicated conditions, coverslips were removed from the dish, washed with PBS, fixed with 4% formaldehyde, permeabilized with 0.2% Triton X-100, washed, and blocked in 0.5% fish gelatin in PBS containing 0.1% Tween 20. EdU was detected using the Click-iT EdU reaction (Thermo Fisher Scientific), and coverslips were washed, incubated with DAPI (D9542; Sigma), and mounted using Vectashield (H-1000; Vector Laboratories). Images were acquired using a CCD camera connected to an Olympus IX70 deltavision deconvolution microscope under a 100× oil immersion objective. Images were analyzed using Volocity 3D Image Analysis software (PerkinElmer). Briefly, each nucleus was outlined as the region of interest utilizing the DAPI signal, and this was used to determine total nuclear EdU. After setting the nuclear background intensity, the number of nuclear EdU foci and intensity of EdU signal were obtained by running the following program: (1) find objects using intensity; (2) clip to region of interests (ROIs); (3) remove noise from objects (fine filter); (4) separate touching objects (object size guide, 0.02 μm^3); (5) exclude objects by size (exclude objects < 0.015 μm^3).

Recombinant Proteins, Reagents, and Antibodies

Full-length $p27^{kip1}$ was expressed from pGEX-$p27^{kip1}$ plasmid (a gift of J. Walter, Harvard Medical School) as described previously (Poh et al., 2014).

Chemicals were acquired as follows: PHA-767491, from the Division of Signal Transduction Therapy, University of Dundee; XL413, Axon Medchem (#2268) and a gift from Cancer Research Technology; tautomycetin, Tocris (#2305); CHIR-124, Axon Medchem (Axon 1636); and KU55933, Axon Medchem (Axon 1367). Antibodies against *Xenopus* proteins were as described: Mcm3, Mcm4, and Mcm7 (Prokhorova and Blow, 2000); Cdc45 and Psf2 (Gambus et al., 2011). *Xenopus* Rif1 antibody was raised in rabbit against the C-terminal 100 amino acids expressed in bacteria; the antisera were purified over protein A beads (see Figure S2). Antibodies against human Mcm4 (sc-22779), Mcm5 (sc-136336), PCNA (sc-56), PP1α (sc-6104), and PP1γ (sc-6108) were from Santa Cruz Biotechnology. Anti-Mcm2 was from BD Biosciences (#610701). Lamin B (ab16048), Mcm2 (phospho S40) (ab133243), Mcm2 (phospho S53) (ab109133), Rif1 (ab140464 and ab70254), and Cdc7 (ab1053) were from Abcam. Tubulin (T6199) was from Sigma-Aldrich. Antibodies raised against human Mcm2 and phospho-Mcm2 recognize the cognate proteins in *Xenopus* (Figures S2B–S2D; note that the *Xenopus* orthologs of human Mcm2 Ser40 and Ser53 are Ser25 and Ser38, respectively).

SUPPLEMENTAL INFORMATION

Supplemental Information includes six figures and can be found with this article online at http://dx.doi.org/10.1016/j.celrep.2017.02.042.

AUTHOR CONTRIBUTIONS

R.C.A. performed all the cell-culture experiments. G.S.C. performed all the *Xenopus* experiments and Rif1 IP from HeLa cells. P.J.G. prepared and G.S.C. characterized the *Xenopus* Rif1 antibody. J.J.B. conceived and supervised the research project. R.C.A., G.S.C., P.J.G., and J.J.B. wrote the manuscript.

ACKNOWLEDGMENTS

We would like to acknowledge the Flow Cytometry and Cell Sorting Facility at the University of Dundee, Dr. Sara ten Have and Kelly Hodge of the GRE Proteomics Support Team at the University of Dundee (Wellcome Trust Strategic Award 097945/Z/11/Z), and Dr. Sara Buonomo and her lab for helpful discussions. This work was funded by Cancer Research UK programme grant C303/A14301, Wellcome Trust Senior Investigator Award WT096598MA, and Wellcome Trust Strategic Award 097945/B/11/Z.

Received: September 16, 2016
Revised: January 5, 2017
Accepted: February 14, 2017
Published March 7, 2017

REFERENCES

Bruck, I., and Kaplan, D.L. (2015). The Dbf4-Cdc7 kinase promotes Mcm2-7 ring opening to allow for single-stranded DNA extrusion and helicase assembly. J. Biol. Chem. *290*, 1210–1221.

Cornacchia, D., Dileep, V., Quivy, J.P., Foti, R., Tili, F., Santarella-Mellwig, R., Antony, C., Almouzni, G., Gilbert, D.M., and Buonomo, S.B. (2012). Mouse Rif1 is a key regulator of the replication-timing programme in mammalian cells. EMBO J. *31*, 3678–3690.

Costanzo, V., Shechter, D., Lupardus, P.J., Cimprich, K.A., Gottesman, M., and Gautier, J. (2003). An ATR- and Cdc7-dependent DNA damage checkpoint that inhibits initiation of DNA replication. Mol. Cell *11*, 203–213.

Davé, A., Cooley, C., Garg, M., and Bianchi, A. (2014). Protein phosphatase 1 recruitment by Rif1 regulates DNA replication origin firing by counteracting DDK activity. Cell Rep. *7*, 53–61.

Foti, R., Gnan, S., Cornacchia, D., Dileep, V., Bulut-Karslioglu, A., Diehl, S., Buness, A., Klein, F.A., Huber, W., Johnstone, E., et al. (2016). Nuclear architecture organized by Rif1 underpins the replication-timing program. Mol. Cell *61*, 260–273.

Gambus, A., Khoudoli, G.A., Jones, R.C., and Blow, J.J. (2011). MCM2-7 form double hexamers at licensed origins in Xenopus egg extract. J. Biol. Chem. *286*, 11855–11864.

Gillespie, P.J., and Blow, J.J. (2010). Clusters, factories and domains: The complex structure of S-phase comes into focus. Cell Cycle *9*, 3218–3226.

Gillespie, P.J., Gambus, A., and Blow, J.J. (2012). Preparation and use of Xenopus egg extracts to study DNA replication and chromatin associated proteins. Methods *57*, 203–213.

Gillespie, P.J., Neusiedler, J., Creavin, K., Chadha, G.S., and Blow, J.J. (2016). Cell cycle synchronization in Xenopus egg extracts. Methods Mol. Biol. *1342*, 101–147.

Hardy, C.F., Dryga, O., Seematter, S., Pahl, P.M., and Sclafani, R.A. (1997). mcm5/cdc46-bob1 bypasses the requirement for the S phase activator Cdc7p. Proc. Natl. Acad. Sci. USA *94*, 3151–3155.

Hayano, M., Kanoh, Y., Matsumoto, S., Renard-Guillet, C., Shirahige, K., and Masai, H. (2012). Rif1 is a global regulator of timing of replication origin firing in fission yeast. Genes Dev. *26*, 137–150.

Hiraga, S., Alvino, G.M., Chang, F., Lian, H.Y., Sridhar, A., Kubota, T., Brewer, B.J., Weinreich, M., Raghuraman, M.K., and Donaldson, A.D. (2014). Rif1 controls DNA replication by directing Protein Phosphatase 1 to reverse Cdc7-mediated phosphorylation of the MCM complex. Genes Dev. *28*, 372–383.

Hughes, S., Elustondo, F., Di Fonzo, A., Leroux, F.G., Wong, A.C., Snijders, A.P., Matthews, S.J., and Cherepanov, P. (2012). Crystal structure of human CDC7 kinase in complex with its activator DBF4. Nat. Struct. Mol. Biol. *19*, 1101–1107.

Jares, P., and Blow, J.J. (2000). Xenopus cdc7 function is dependent on licensing but not on XORC, XCdc6, or CDK activity and is required for XCdc45 loading. Genes Dev. *14*, 1528–1540.

Jiang, W., McDonald, D., Hope, T.J., and Hunter, T. (1999). Mammalian Cdc7-Dbf4 protein kinase complex is essential for initiation of DNA replication. EMBO J. *18*, 5703–5713.

Koltun, E.S., Tsuhako, A.L., Brown, D.S., Aay, N., Arcalas, A., Chan, V., Du, H., Engst, S., Ferguson, K., Franzini, M., et al. (2012). Discovery of XL413, a potent and selective CDC7 inhibitor. Bioorg. Med. Chem. Lett. *22*, 3727–3731.

Kumar, R., and Cheok, C.F. (2014). RIF1: a novel regulatory factor for DNA replication and DNA damage response signaling. DNA Repair (Amst.) *15*, 54–59.

Lei, M., Kawasaki, Y., Young, M.R., Kihara, M., Sugino, A., and Tye, B.K. (1997). Mcm2 is a target of regulation by Cdc7-Dbf4 during the initiation of DNA synthesis. Genes Dev. *11*, 3365–3374.

Luciani, M.G., Oehlmann, M., and Blow, J.J. (2004). Characterization of a novel ATR-dependent, Chk1-independent, intra-S-phase checkpoint that suppresses initiation of replication in Xenopus. J. Cell Sci. *117*, 6019–6030.

Masai, H., Matsui, E., You, Z., Ishimi, Y., Tamai, K., and Arai, K. (2000). Human Cdc7-related kinase complex. In vitro phosphorylation of MCM by concerted actions of Cdks and Cdc7 and that of a criticial threonine residue of Cdc7 bY Cdks. J. Biol. Chem. *275*, 29042–29052.

Masai, H., Taniyama, C., Ogino, K., Matsui, E., Kakusho, N., Matsumoto, S., Kim, J.M., Ishii, A., Tanaka, T., Kobayashi, T., et al. (2006). Phosphorylation of MCM4 by Cdc7 kinase facilitates its interaction with Cdc45 on the chromatin. J. Biol. Chem. *281*, 39249–39261.

Mattarocci, S., Shyian, M., Lemmens, L., Damay, P., Altintas, D.M., Shi, T., Bartholomew, C.R., Thomä, N.H., Hardy, C.F., and Shore, D. (2014). Rif1 controls DNA replication timing in yeast through the PP1 phosphatase Glc7. Cell Rep. *7*, 62–69.

Montagnoli, A., Bosotti, R., Villa, F., Rialland, M., Brotherton, D., Mercurio, C., Berthelsen, J., and Santocanale, C. (2002). Drf1, a novel regulatory subunit for human Cdc7 kinase. EMBO J. *21*, 3171–3181.

Montagnoli, A., Valsasina, B., Brotherton, D., Troiani, S., Rainoldi, S., Tenca, P., Molinari, A., and Santocanale, C. (2006). Identification of Mcm2 phosphorylation sites by S-phase-regulating kinases. J. Biol. Chem. *281*, 10281–10290.

Montagnoli, A., Valsasina, B., Croci, V., Menichincheri, M., Rainoldi, S., Marchesi, V., Tibolla, M., Tenca, P., Brotherton, D., Albanese, C., et al.

(2008). A Cdc7 kinase inhibitor restricts initiation of DNA replication and has antitumor activity. Nat. Chem. Biol. *4*, 357–365.

O'Keefe, R.T., Henderson, S.C., and Spector, D.L. (1992). Dynamic organization of DNA replication in mammalian cell nuclei: spatially and temporally defined replication of chromosome-specific alpha-satellite DNA sequences. J. Cell Biol. *116*, 1095–1110.

Poh, W.T., Chadha, G.S., Gillespie, P.J., Kaldis, P., and Blow, J.J. (2014). Xenopus Cdc7 executes its essential function early in S phase and is counteracted by checkpoint-regulated protein phosphatase 1. Open Biol. *4*, 130138.

Prokhorova, T.A., and Blow, J.J. (2000). Sequential MCM/P1 subcomplex assembly is required to form a heterohexamer with replication licensing activity. J. Biol. Chem. *275*, 2491–2498.

Renard-Guillet, C., Kanoh, Y., Shirahige, K., and Masai, H. (2014). Temporal and spatial regulation of eukaryotic DNA replication: from regulated initiation to genome-scale timing program. Semin. Cell Dev. Biol. *30*, 110–120.

Sasi, N.K., Tiwari, K., Soon, F.F., Bonte, D., Wang, T., Melcher, K., Xu, H.E., and Weinreich, M. (2014). The potent Cdc7-Dbf4 (DDK) kinase inhibitor XL413 has limited activity in many cancer cell lines and discovery of potential new DDK inhibitor scaffolds. PLoS ONE *9*, e113300.

Sato, N., Arai, K., and Masai, H. (1997). Human and Xenopus cDNAs encoding budding yeast Cdc7-related kinases: in vitro phosphorylation of MCM subunits by a putative human homologue of Cdc7. EMBO J. *16*, 4340–4351.

Sheu, Y.J., and Stillman, B. (2006). Cdc7-Dbf4 phosphorylates MCM proteins via a docking site-mediated mechanism to promote S phase progression. Mol. Cell *24*, 101–113.

Sheu, Y.J., and Stillman, B. (2010). The Dbf4-Cdc7 kinase promotes S phase by alleviating an inhibitory activity in Mcm4. Nature *463*, 113–117.

Smits, V.A., and Gillespie, D.A. (2015). DNA damage control: regulation and functions of checkpoint kinase 1. FEBS J. *282*, 3681–3692.

Sørensen, C.S., Hansen, L.T., Dziegielewski, J., Syljuåsen, R.G., Lundin, C., Bartek, J., and Helleday, T. (2005). The cell-cycle checkpoint kinase Chk1 is required for mammalian homologous recombination repair. Nat. Cell Biol. *7*, 195–201.

Sreesankar, E., Senthilkumar, R., Bharathi, V., Mishra, R.K., and Mishra, K. (2012). Functional diversification of yeast telomere associated protein, Rif1, in higher eukaryotes. BMC Genomics *13*, 255.

Takahashi, T.S., and Walter, J.C. (2005). Cdc7-Drf1 is a developmentally regulated protein kinase required for the initiation of vertebrate DNA replication. Genes Dev. *19*, 2295–2300.

Walter, J.C. (2000). Evidence for sequential action of cdc7 and cdk2 protein kinases during initiation of DNA replication in Xenopus egg extracts. J. Biol. Chem. *275*, 39773–39778.

Woodward, A.M., Göhler, T., Luciani, M.G., Oehlmann, M., Ge, X., Gartner, A., Jackson, D.A., and Blow, J.J. (2006). Excess Mcm2-7 license dormant origins of replication that can be used under conditions of replicative stress. J. Cell Biol. *173*, 673–683.

Yamazaki, S., Ishii, A., Kanoh, Y., Oda, M., Nishito, Y., and Masai, H. (2012). Rif1 regulates the replication timing domains on the human genome. EMBO J. *31*, 3667–3677.

Yanow, S.K., Gold, D.A., Yoo, H.Y., and Dunphy, W.G. (2003). Xenopus Drf1, a regulator of Cdc7, displays checkpoint-dependent accumulation on chromatin during an S-phase arrest. J. Biol. Chem. *278*, 41083–41092.

Yoshizawa-Sugata, N., Ishii, A., Taniyama, C., Matsui, E., Arai, K., and Masai, H. (2005). A second human Dbf4/ASK-related protein, Drf1/ASKL1, is required for efficient progression of S and M phases. J. Biol. Chem. *280*, 13062–13070.

Cell Reports, Volume *18*

Supplemental Information

Reversal of DDK-Mediated MCM Phosphorylation by Rif1-PP1 Regulates Replication Initiation and Replisome Stability Independently of ATR/Chk1

Robert C. Alver, Gaganmeet Singh Chadha, Peter J. Gillespie, and J. Julian Blow

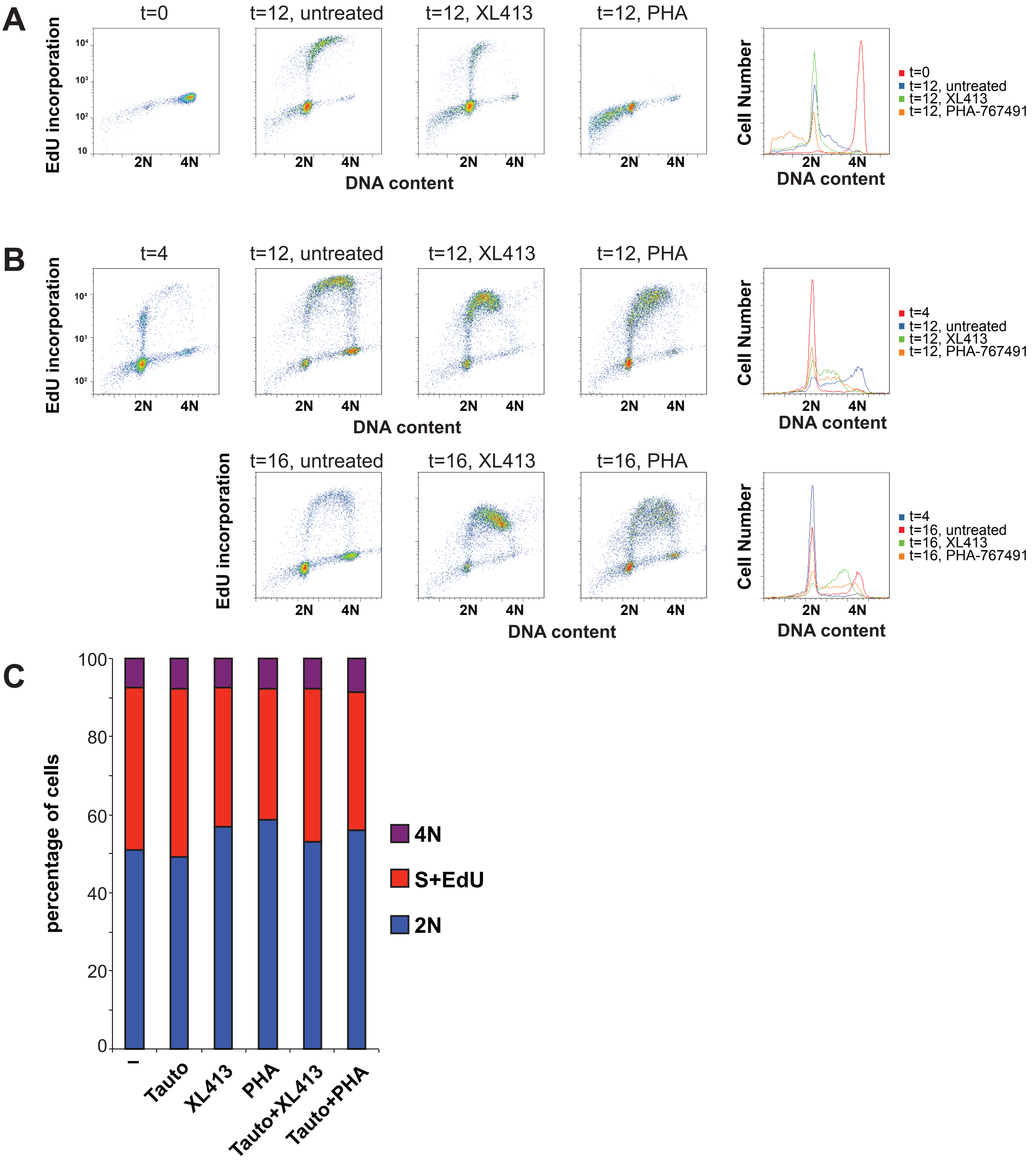

Figure S1. Effect of PP1 and Cdc7 inhibitors on the cell cycle. ***Related to Figure 2***

A-B, HeLa cells were synchronized by mitotic shakeoff and were (A) immediately treated for 12 hrs with 10 µM PHA-767491 or 10 µM XL413 or (B) treated with the same concentration of Cdc7 inhibitor for 8 or 12 hours after allowing cells to move into early S phase. Cells were pulsed with EdU for 30 min and analysed by flow cytometry. 2D EdU/DNA content plots are shown for each treatment. Frequency plots of DNA content are shown on the right. Note that the plots have not had the sub-2N population gated out.

C, HeLa cells were synchronized by mitotic shakeoff, allowed to move into early S phase and then subsequently treated for 2 hours with 225 nM tautomycetin, 10 µM XL413, or 10 µM PHA-767491 in the indicated combinations. The relative populations of cells in each cell cycle phase were quantified by 2D flow cytometry.

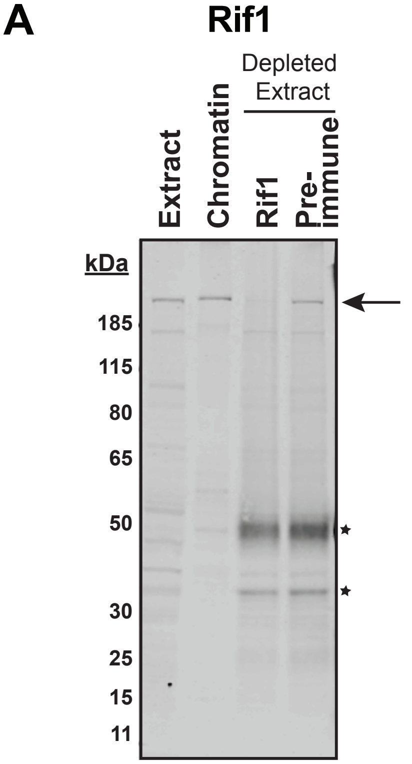

B

Xenopus

Protein Description	Peptide Count	
	Rif1	IgG
1. Rif1 protein	81	0
2. Pcm-1 protein; Pericentriolar material 1 protein	52	0
3. Nucleoprotein TPR (Fragment)	19	0
4. plectin-like isoform X3	6	0
5. Ataxia telangiectasia mutated (Fragment); ATM	3	0
6. CAP-GLY domain containing linker protein 1	3	0
7. Haspin	3	0

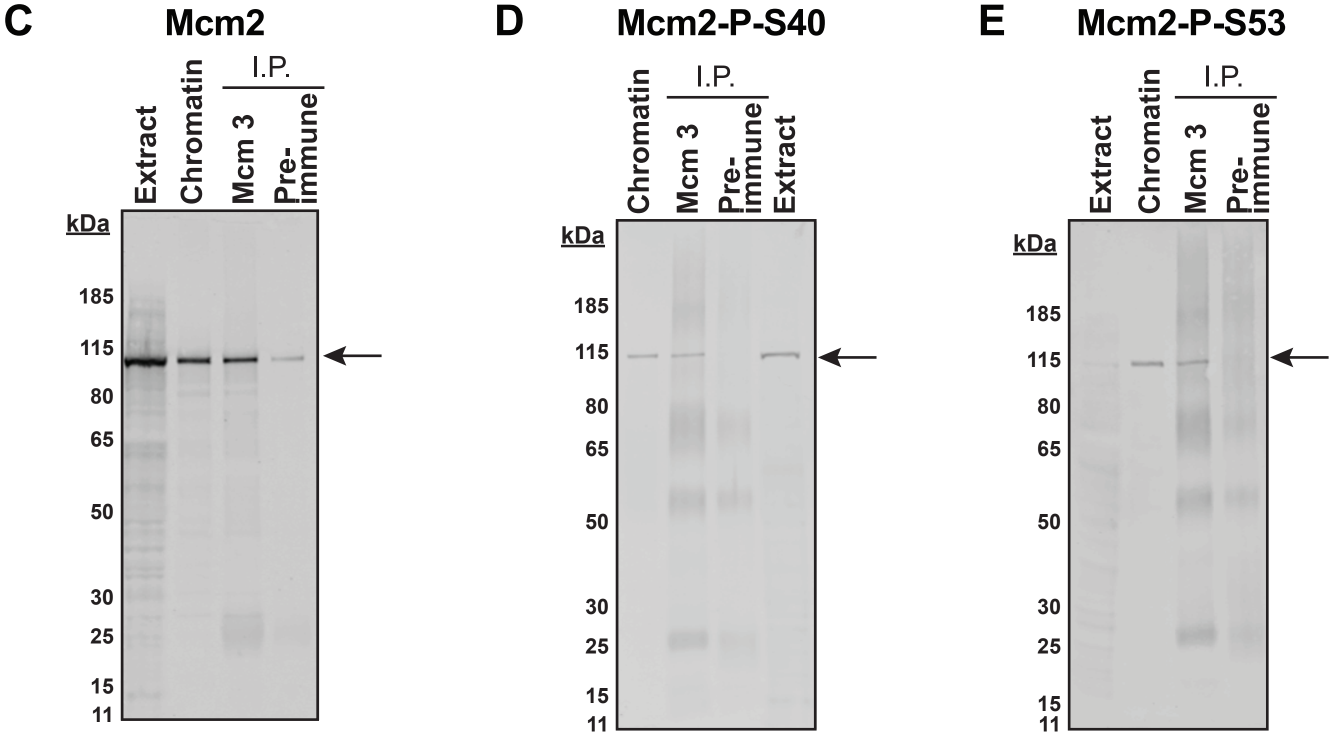

Figure S2. **Characterisation of antibodies in Xenopus.** ***Related to Figure 3***
A, 0.5 µl Xenopus egg extract, chromatin isolated from early S phase (40 minutes) and 0.5 µl extract depleted using antibodies against Rif1 or pre-immune IgGs were separated by SDS-PAGE and immunoblotted with the Xenopus Rif1 antibody. **B**, Immunoprecipitations (IP) from Xenopus egg extract using Rif1 antibody or control rabbit IgG (Sigma) covalently coupled to Protein-A Sepharose beads. IP samples were run on a 4–12% gradient NuPAGE gel (Invitrogen). The gel was stained with SimplyBlue SafeStain (Invitrogen). Bands at the size of Rif1 were cut from both Rif1 and IgG lanes, samples were reduced with dithiothreitol, alkylated with iodoacetamide and in-gel digested with trypsin. The extracted peptide solutions were analysed using nano LC-MS/MS on an LTQ Orbitrap Velos (ThermoFisher, San Jose, CA). The top five hits by peptide number for proteins identified only in Rif1 IP and not in control IgG IP are presented. **C-E**, 0.5 µl Xenopus egg extract, chromatin isolated from early S phase (40 minutes) and proteins immunoprecipitated using Xenopus Mcm3 or pre-immune serum were separated by SDSPAGE and immunoblotted with the Xenopus Mcm2 (C), Mcm2-P-S40 (D) and Mcm2-P-53 (E) antibody. The migration of molecular weight markers (sizes in kDa) is shown to the left.

Xenopus

A

Protein Description	Peptide Count	
	**Rif1**	**IgG**
Rif1 protein	48	0
Serine/threonine-protein phosphatase PP1-beta catalytic subunit;ppp1cb	3	0

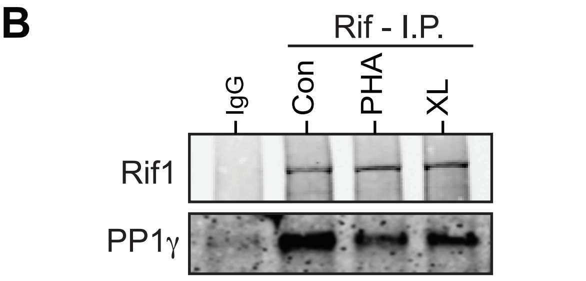

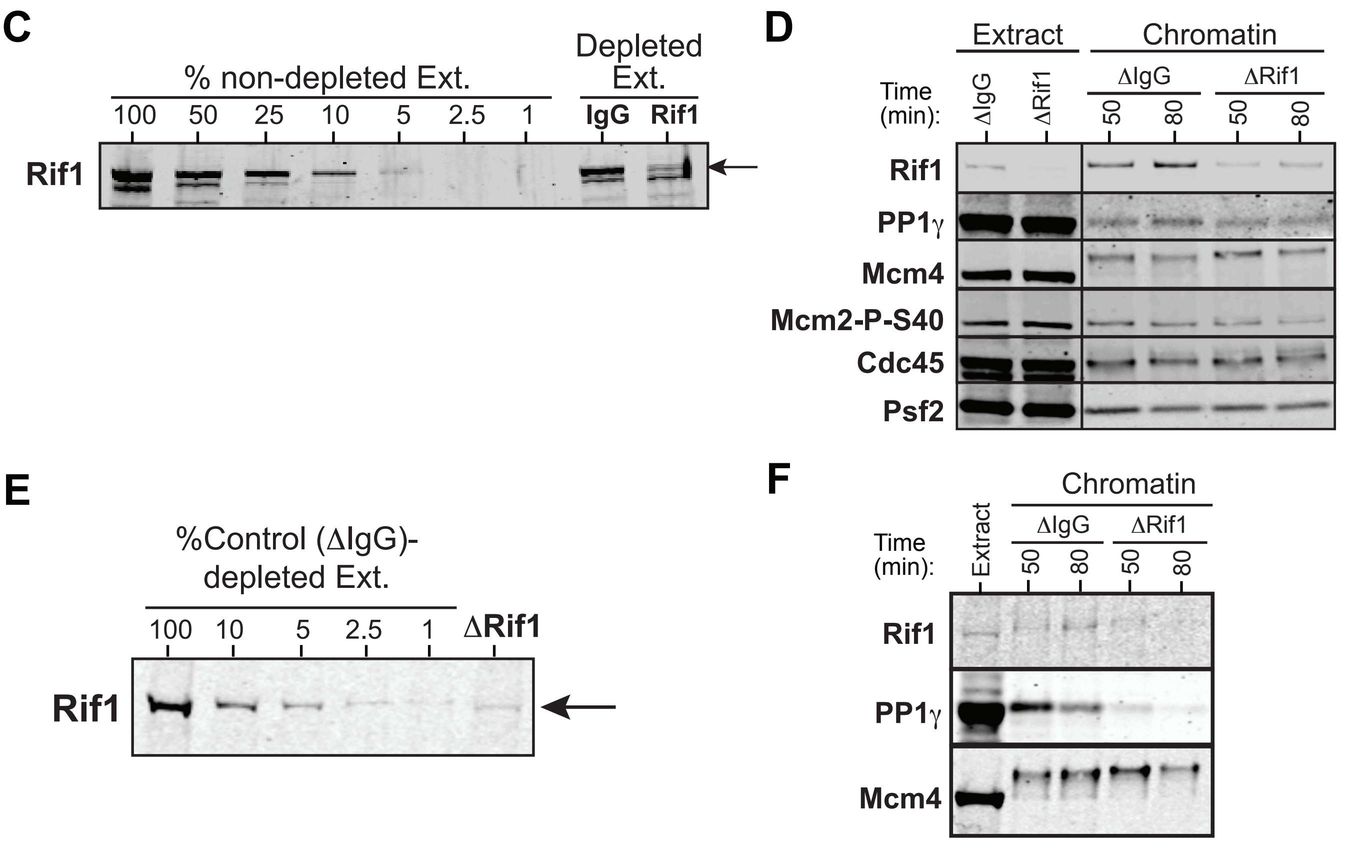

Figure S3. Characterisation of Rif1 depletion in Xenopus egg extract. ***Related to Figure 3***
A, Chromatin was isolated from Xenopus egg extract in mid S-phase. Immunoprecipitations (IP) using Xenopus Protein-A-purified Rif1 antibody or control non-immune IgG coupled to Protein-A Sepharose was carried out as described (Gillespie et al. 2012). The samples were reduced with dithiothreitol, alkylated with iodoacetamide and digested with trypsin. The extracted peptide solutions were analysed using nano LC-MS/MS on an LTQ Orbitrap Velos (ThermoFisher, San Jose, CA). The top hits by peptide number for proteins identified only in Rif1 IP and not in control IgG IP are presented. **B**, Xenopus egg extract were optionally supplemented and incubated with PHA-767491 or XL413 for 45 min before Rif1 was immunoprecipitated from extracts using antibodies against Rif1 or with pre-immune rabbit IgG. The samples were immunoblotted for Rif1 and PP1γ. **C-F**, Xenopus egg extract was immunodepleted with either nonimmune IgG or Rif1 antibodies. Two different extent of depletions are shown (C,D and E,F). So that the efficiency of depletion could be estimated, 0.5 µl of each of the depleted extracts and known amounts of control depleted extract was immunoblotted for Rif1. The efficiency of immunodepletion was >90% (C) and >95% (E). D, F, Control (ΔIgG) and Rif1 (ΔRif1) depleted Xenopus extracts from C and E were incubated with demembranated sperm nuclei. Chromatin was isolated at indicated times and samples were immunoblotted for Rif1, Mcm4, Mcm2-P-S40, Cdc45, Psf2, and PP1γ.

Xenopus

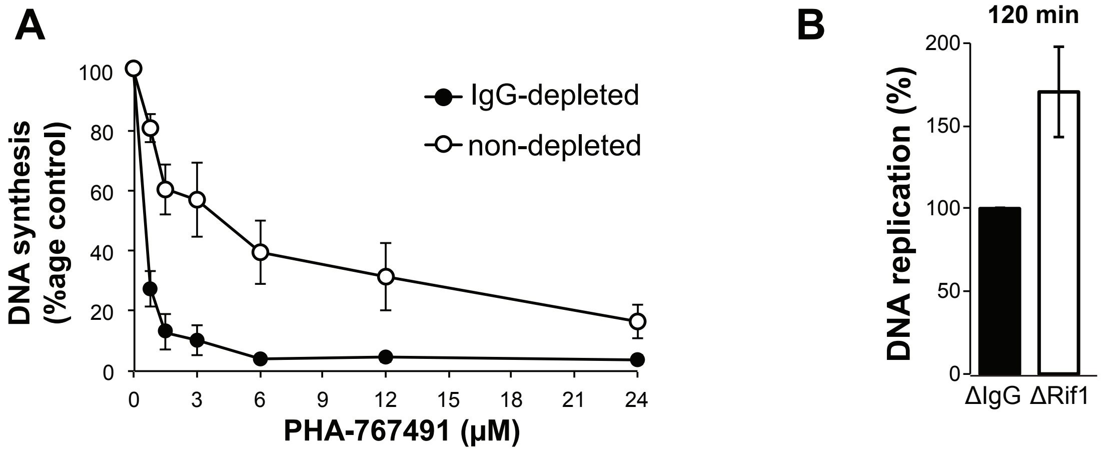

Figure S4. DNA replication upon Rif1 depletion in Xenopus egg extract. ***Related to Figure 3 & 4***
A, Control (non-depleted) or IgG-depleted Xenopus extracts were incubated with 3 ng/μl sperm DNA, [α-^{32}P]dATP and increasing concentration of PHA-767491. Total DNA synthesis at 240 min is expressed as a percentage of the synthesis in the absence of PHA-767491. Mean and S.E.M. of three independent experiments is shown. **B**, Control (Δ IgG) and Rif1 (ΔRif1) depleted Xenopus extracts were incubated with demembranated sperm nuclei and [α-^{32}P]dATP. Total DNA synthesis at 120 min is expressed as a percentage of the synthesis in control (ΔIgG-depleted) extract. Mean and S.E.M. of three independent experiments is shown.

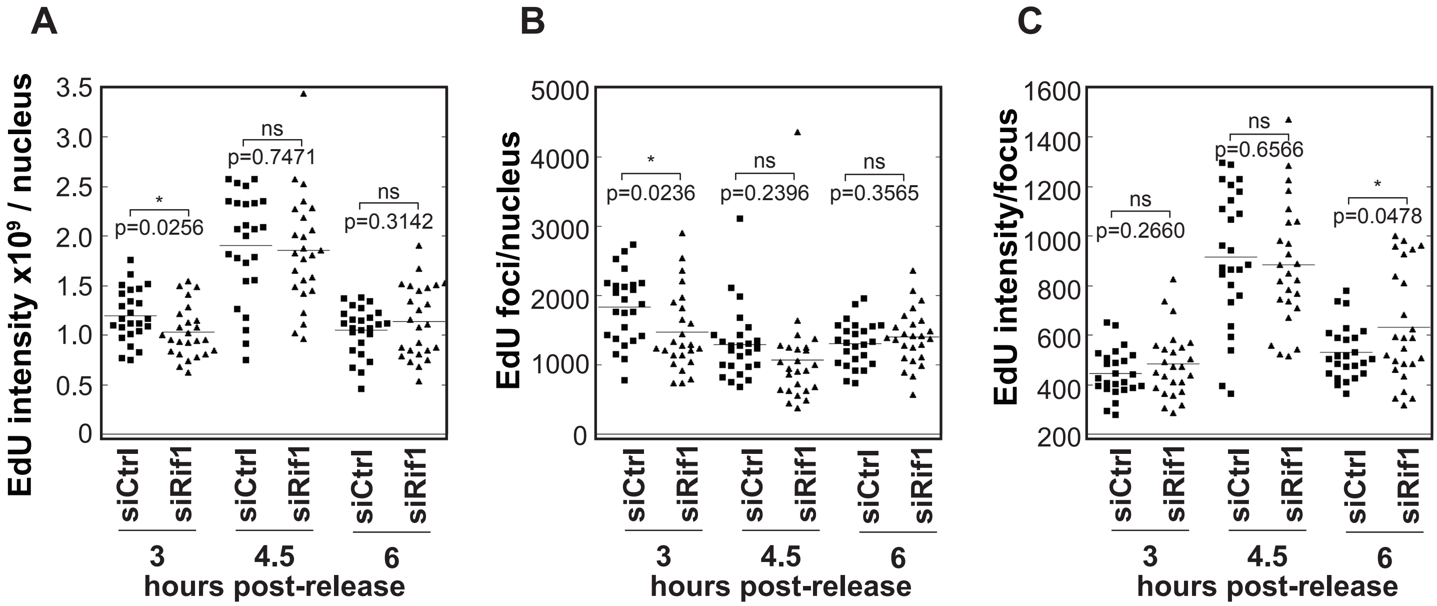

Figure S5. Quantification of the 3, 4.5, and 6 hr timepoints from Figure 5

HeLa cells were treated with control or Rif1 siRNA, synchronized by double thymidine block and then released. At the indicated periods after release cells were pulsed with EdU for 30 min. The spatial patterns of DNA synthesis were examined by fluorescence microscopy. At the indicated times after release the total EdU intensity of the nucleus (**A**), the number of replication foci per nucleus (**B**) and the intensity of the EdU foci (**C**) were quantified (25 nuclei/condition).

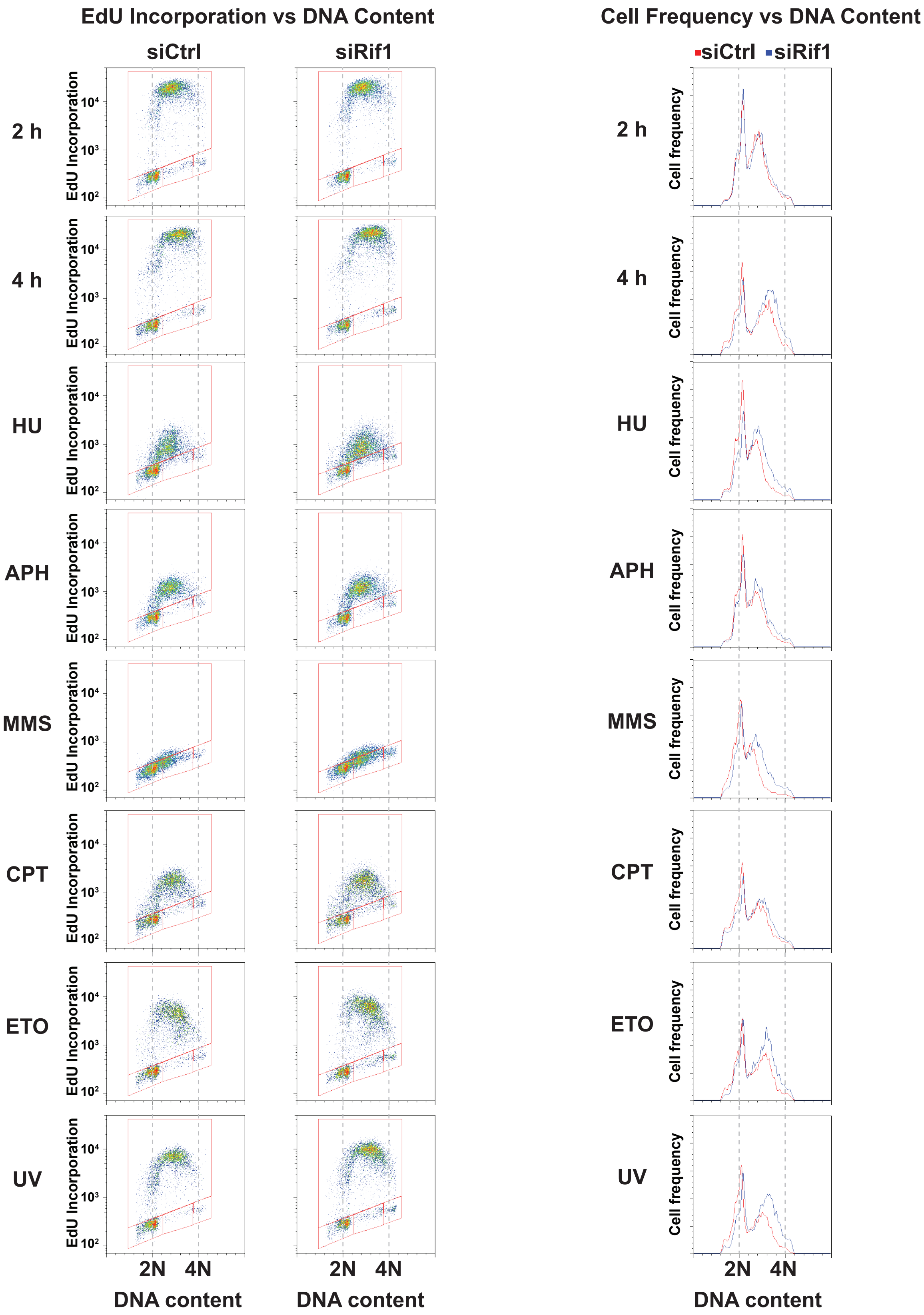

Figure S6. Rif1 regulates S phase progression and replisome stability. ***Related to Figure 7***
This is the same experiment as Fig. 7C. HeLa cells were treated control or Rif1 siRNA, synchronized by double thymidine block, and released 2 hrs before either no treatment or after the addition of hydroxyurea (5 mM), aphidicolin (1 mg/mL), methyl methanesulfonate (0.02%), camptothecin (5 mM) etoposide (5 mM), or treating cells with UV irradiation (~45 J/m^2). Chemical treatments and recovery from irradiation lasted two hours before cells were harvested, fixed and subsequently analysed by flow cytometry. 2D EdU/DNA content plots are shown for each treatment. Frequency plots of DNA content are shown on the right.
